# Physical activity and perinatal depression and anxiety: a global umbrella review and meta-meta-analysis

**DOI:** 10.1016/j.eclinm.2026.104054

**Published:** 2026-07-19

**Authors:** Elizabeth Mohr, Helena Ludwig-Walz, Dawid Pieper, Regina Guthold, Mariebelle Kaus, Alexander Pachanov, Martin Bujard

**Affiliations:** a Federal Institute for Population Research (BiB), Wiesbaden, Germany; b Institute of Medical Psychology, Medical Faculty, Heidelberg University, Heidelberg, Germany; c Institute for Health Services and Health System Research, Faculty of Health Sciences Brandenburg, Brandenburg Medical School (Theodor Fontane), Rüdersdorf, Germany; d Center for Health Services Research, Brandenburg Medical School (Theodor Fontane), Rüdersdorf, Germany; e Department of Sexual, Reproductive, Maternal, Child and Adolescent and Ageing Health, World Health Organization, Geneva, Switzerland; f Institute of Social Medicine and Health Systems Research, University of Magdeburg, Magdeburg, Germany

**Keywords:** Maternal mental health, Perinatal mental health, Physical activity, Anxiety, Depression, Umbrella review

## Abstract

**Background:**

Physical activity is increasingly recognized as a non-pharmacological intervention reducing the risk of perinatal depression and anxiety, but findings from previous meta-analyses are heterogeneous. This umbrella review synthesized evidence on the overall effects of physical activity on perinatal depression and anxiety in women, including by activity intensity and type.

**Methods:**

We searched PubMed, Embase, Web of Science, Cochrane Library, PsycINFO, Epistemonikos, DoPHER, CINAHL, Sports Medicine & Education Index for literature published between Jan 2014 and April 2026. Eligible systematic reviews assessed randomized controlled trials of the effect of physical activity interventions on perinatal depression and/or anxiety outcomes. Pairs of authors conducted data extraction, methodological quality (AMSTAR-2) and certainty of evidence (GRADE) assessments. Overlap between included systematic reviews was assessed using the corrected covered area. Random-effects meta-meta-analyses (a meta-analysis of meta-analyses) yielded standardized mean differences (SMDs) with 95% confidence intervals (95% CIs). PROSPERO registry: CRD42024538941.

**Findings:**

A total of 4109 studies were retrieved and 20 systematic reviews with meta-analyses were included. Overlap was 8.49%. Overall physical activity reduces depressive symptoms during the perinatal period with moderate certainty of evidence (SMD: −0.56; 95%-CI: −0.93 to −0.19). Light-intensity physical activity (SMD: −0.52; 95% CI: −0.81 to −0.23) may have effects comparable to those of moderate (SMD: −0.43; 95% CI: −0.64 to −0.22) and mixed-intensity (SMD: −0.52; 95% CI: −0.80 to −0.24) interventions. Yoga (SMD: −0.55; 95%-CI: −0.95 to −0.15) and mixed activity (SMD: −0.54; 95%-CI: −0.70 to −0.38) may be particularly beneficial. PA may also be effective in reducing anxiety (SMD: −0.85, 95%-CI: −1.29 to −0.42), though research is more limited.

**Interpretation:**

Light and moderate physical activity are positively associated with a reduction in perinatal depressive and anxiety symptoms in women. Physical activity should be recommended as a non-pharmacological, feasible, and cost-effective approach to address perinatal depression, and potentially anxiety, globally.

**Funding:**

None.

**Research in context**

**Evidence before this study**

We conducted an extensive literature search across multiple databases (PubMed, Embase, Web of Science, Cochrane Library, PsycINFO, Epistemonikos, DoPHER) to identify previous systematic reviews and meta-analyses on non-pharmacological interventions, for perinatal depression and anxiety. This preliminary search suggested that physical activity may be a potential intervention for perinatal depression and anxiety. Although several reviews and meta-analyses have examined different types of physical activity interventions, the evidence remains heterogeneous, with inconsistent findings regarding their effects on perinatal depression and anxiety and the characteristics of effective interventions, including activity type and intensity. We identified two previous meta-meta-analyses of physical activity in the general adult population that reported small to moderate reductions in depression and anxiety, with the more recent study concluding that high overlap warrants further studies focusing specifically on the perinatal population.

**Added value of this study**

To our knowledge, this is the first umbrella review with meta-meta-analysis to provide a comprehensive global synthesis of the effect of physical activity interventions on perinatal depressive and anxiety symptoms. Findings from our meta-meta-analysis show that physical activity is associated with a small-to-moderate reduction in perinatal depressive symptoms. Specifically, our estimates show that light-, moderate- and mixed-intensity physical activity is effective in reducing perinatal depressive symptoms. In terms of specific interventions, yoga and mixed activities may be particularly effective. We were also able to synthesize effect estimates for prenatal anxiety symptoms, suggesting that yoga may be effective, but more research on perinatal anxiety and physical activity is needed.

**Implications of all the available evidence**

The results of this umbrella review with meta-meta-analysis indicate that physical activity is an essential public health intervention to promote perinatal mental health globally. Our findings highlight the importance of incorporating physical activity into health systems, communities, and public health policy in order to promote the physical and mental health of mothers. Based on these findings, we suggest that research and practice should shift the focus to developing and implementing physical activity interventions that are adapted to the needs and contexts of perinatal populations. This includes expanding current guidelines on physical activity and perinatal mental health to highlight the effectiveness of light-intensity physical activity alongside moderate activities and adding more concrete recommendations (e.g., yoga for perinatal depressive symptoms) for implementing physical activity interventions in the perinatal context. To increase awareness, we also recommend integrating information on the link between physical activity and perinatal mental health into health care provider training and public health campaigns. Future research should focus on interventions targeted specifically at perinatal anxiety and on effective implementation in settings with limited resources.

## Introduction

Globally, around 10% of pregnant and 13% of postpartum women experience a mental health problem,^1^ most commonly depression and anxiety.^2^ Perinatal depression and anxiety have implications for women, their children, and their families,^2^^,^^3^ making preventing and treating perinatal mental health disorders an important public health issue. Standard interventions for perinatal depression and anxiety may include psychotropic drugs and psychotherapy.^2^ However, concerns about side effects of medication use during pregnancy^4^ and barriers to accessing psychotherapy^5^ risks leaving these conditions undertreated. Umbrella evidence for the general population indicates that physical activity (PA) reduces symptoms of depression and anxiety^6^ and recent systematic reviews with meta-analysis suggest potential benefits for depressive and anxiety symptoms in perinatal populations.^7^, ^8^, ^9^

However, while promising, findings across these existing reviews are inconsistent. Firstly, it remains unclear whether overall PA has small,^8^ moderate^7^^,^^9^, ^10^, ^11^ or large^12^ effects on perinatal depressive symptoms. Secondly, findings on effective PA types are inconsistent, for example, yoga for prenatal depressive symptoms (significant^13^ or non-significant^10^). Thirdly, it is unclear which PA intensity is most effective in reducing depressive symptoms, with some findings supporting low-intensity PA for prenatal depressive symptoms^14^ and other studies suggesting that moderate to high-intensity PA is effective for postpartum depression.^7^^,^^15^ Fourth, evidence on the effect of PA on perinatal anxiety symptoms is inconclusive.^9^^,^^16^ In sum, key evidence gaps include variability in effect size, scope, measurement tools, quality and methodological rigor.

The World Health Organization (WHO) recommends at least 150 min of moderate-intensity aerobic PA per week during pregnancy and postpartum.^17^ Despite this guidance, many women do not meet this target^18^ and health care providers often still report uncertainty regarding appropriate PA guidance.^19^ Two previous meta-meta-analyses of PA in the general adult population reported small to moderate reductions in depression and anxiety,^20^^,^^21^ with the more recent study concluding that high overlap warrants further studies focusing specifically on the perinatal population.^21^ Addressing this call, we conducted an umbrella review of systematic reviews with meta-meta-analysis (a meta-analysis of meta-analyses/second order meta-analysis) of randomized controlled trials (RCTs) to estimate the impact of PA on perinatal depression and anxiety, overall and by PA intensity and type.

## Methods

### Search strategy and selection criteria

For this umbrella review with meta-meta-analysis, we searched MEDLINE, Embase, Web of Science, PsycINFO, Epistemonikos, DoPHER, CINAHL, Cochrane Library, and Sports & Medicine Education Index for studies published in English or German between Jan 2014 and April 2026. Google scholar and reference lists were manually searched. Eligibility criteria followed the population-intervention-comparison-outcome (PICO) framework: Population: women in the perinatal period; Intervention: PA interventions; Comparison: any non-PA comparison; Outcomes: depression and/or anxiety outcomes measured with mostly validated tools or clinical diagnoses. Reviews involving pharmaceutical or psychotherapeutic co-interventions or women with health issues other than depression and anxiety were excluded. Only systematic reviews of RCTs meeting criteria derived from the Cochrane handbook^22^ were included (Supplementary Text S1); those with both observational studies and RCTs were eligible only if RCTs were analyzed separately. The PICO table, complete search strategy, and reasons for exclusion can be found in Supplementary Tables S1–S3.

Screening of titles and abstracts and, subsequently, full-texts was done by two researchers (EM, HLW) using eligibility criteria and *EppiReviewer Software, Version: 6.16.3.0*. Any conflicts over inclusion were resolved between the two researchers (EM, HLW) by comparing reasons for inclusion or exclusion against the pre-defined eligibility criteria and by discussing discrepancies until consensus was reached.

Reviews did not consistently differentiate between symptoms and clinical diagnosis in their assessment; this article refers to depression and depressive symptoms as ‘depressive symptoms’ and anxiety and anxiety symptoms as ‘anxiety symptoms’.

The study was registered on PROSPERO (CRD42024538941) and followed the Preferred Reporting Items for Overviews of Reviews (PRIOR) checklist for overviews of reviews^23^ (Supplementary Table S4), guidance by Fusar-Poli and Radua^24^ (Supplementary Table S5) and The Cochrane Handbook for Systematic Reviews and Interventions.^25^ Deviations from the protocol are reported in Supplementary Table S6.

### Data analysis

Data was extracted and recorded in Excel by one researcher (EM) and checked by another researcher (AP, MK, HLW) and conflicts were resolved through discussion between the researchers. Authors of included papers were contacted about missing data; one review^26^ was excluded because one RCT included in the meta-analysis could not be identified, preventing the verification of primary study details. We extracted data on study characteristics (author name, publication title and year, country of origin of included studies), participants (sample size, age, mental health state), interventions (type, assessment type, timing, session frequency, duration, intensity), and the comparison. For the outcomes, depressive symptoms and anxiety symptoms, we extracted the outcome type and the measurement tool (self-report or diagnosis). We also extracted the risk of bias reported in the study and the results of the meta-analyses and subgroup analyses.

Risk of bias was independently assessed by three researchers in pairs of two (EM, AP, MP) using the ‘A MeaSurement Tool to Assess systematic Reviews-2’ (AMSTAR-2) tool, which includes 16 items and rates overall confidence from ‘critically low’ to ‘high’^27^ (Supplementary Text S2; Supplementary Table S7).

We conducted meta-meta-analyses for overall PA, PA intensity and type based on statistical guidelines from the Cochrane Handbook for Systematic Reviews and Interventions^25^ as meta-meta-analysis is recommended to follow standard approaches used for meta-analyses.^25^ Our rationale for conducting a meta-meta-analysis can be found in Supplementary Text S3.

Prior to data synthesis, we assessed overlap among meta-analyses using the Corrected Covered Area (CCA) approach^28^ (details in Supplementary Text S4; Supplementary Table S8). For overall PA, first-order meta-analyses were categorized by intervention timing (prenatal, postnatal, perinatal PA) and outcome measurement (prenatal, postnatal depressive or anxiety symptoms) and, for subgroup analyses, by light, moderate, vigorous, or mixed intensity based on the 2024 Compendium of Adult Physical Activities^29^ (Supplementary Table S9) and by PA type based on individual meta-analyses. We then assessed overlap between meta-analyses in each category.

When multiple meta-analyses were available and overlap was below 10%, all available meta-analyses were pooled. If only one meta-analysis was available or overlap exceeded 10%, the best available meta-analysis was selected based on methodological quality (e.g., based on number of included RCTs in the review) or publication date.^11^^,^^14^^,^^28^ This hybrid approach was adopted to minimize overlap-related imprecision while retaining as much evidence as possible.^30^

Effect sizes from individual meta-analysis were pooled using the package ‘meta’ in R Studio 4.3.3 and the random effects model, using the standardized mean difference (SMD) and the 95% confidence interval (95%-CI). For PA intensity and PA type, some categories only had a representative systematic review with meta-analysis, so the effect size from this individual meta-analysis was compared with the pooled effects from other categories. Following Cochrane guidelines,^25^ when at least three reviews could be pooled, meta-meta-analysis was performed using the Hartung-Knapp-Sidik-Jonkman method; meta-meta-analyses with two reviews were pooled using the Wald-type method for calculating the 95% CI. Heterogeneity was assessed using forest plots and the I^2^ statistic and sensitivity analyses were conducted for PA intensity and type. For sensitivity analyses, instead of pooling meta-analyses from the 16 eligible reviews, we only pooled meta-analyses used in the meta-meta-analysis for overall effect. This allowed us to compare effect estimates.

Reporting bias was assessed using funnel plots and the Egger's test, though results should be interpreted cautiously given their limited use in umbrella reviews. Certainty of evidence for perinatal depressive symptoms was evaluated independently by two researchers (EM, HLW), using the ‘Grading of Recommendations Assessment, Development, and Evaluation’ (GRADE) approach.^25^ Assessments were based on the information in the systematic reviews, not primary studies. Further details on applying GRADE to umbrella reviews are provided in Supplementary Text S5 and GRADE assessments reported in the systematic reviews are summarized in Supplementary Table S10.

### Role of the funding source

There was no funding source for this study.

## Results

**Fig. 1:**
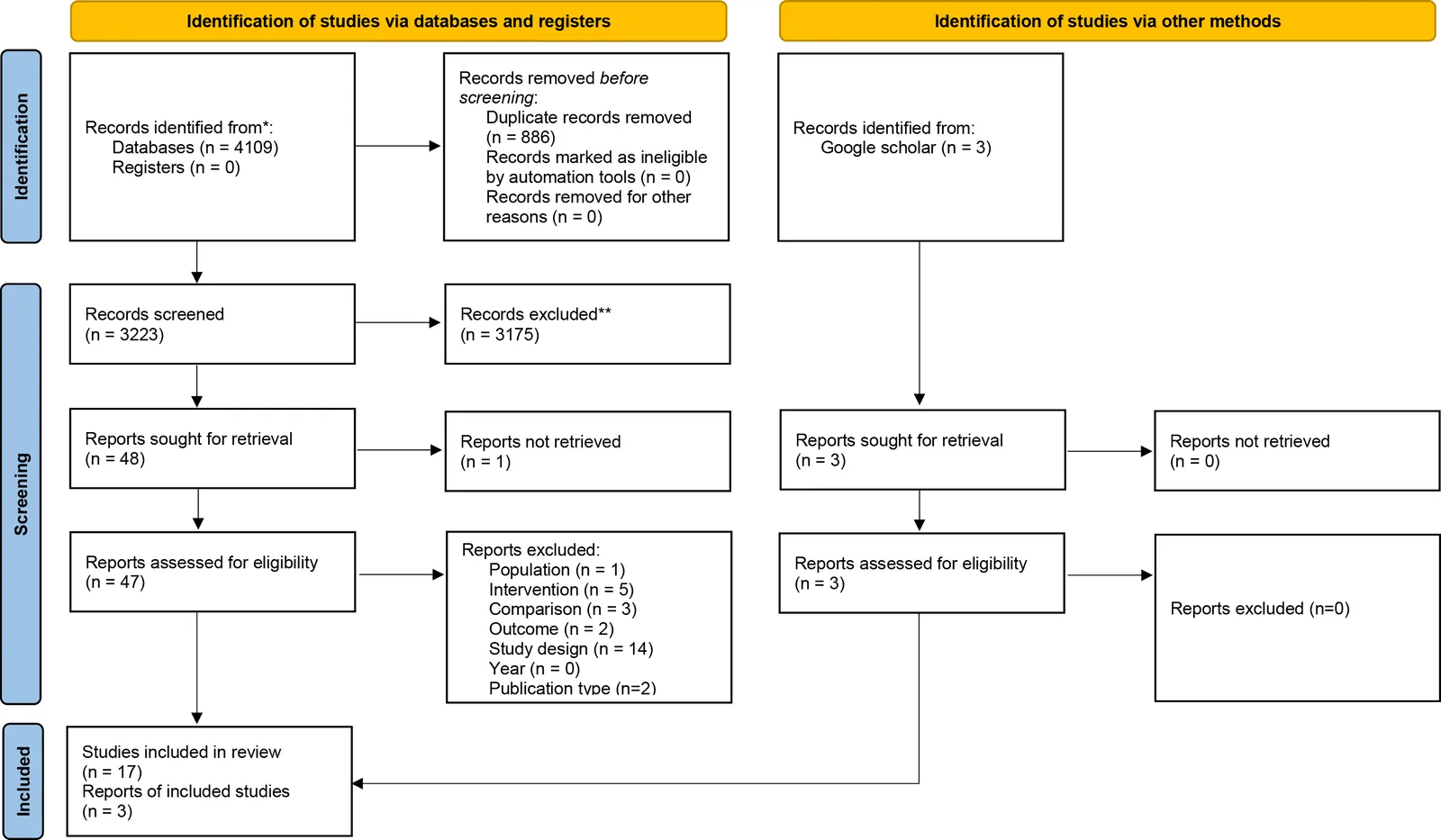
PRISMA 2020 flow diagram for new systematic reviews which included searches of databases, registers and other sources. Source: Page MJ et al. BMJ 2021; 372:n71. https://doi.org/10.1136/bmj.n71.

Out of a total of 4109 identified records, 47 underwent full-text screening and 20 systematic reviews with meta-analysis were included in the final umbrella review. Nineteen assessed depressive symptoms and five anxiety symptoms. See Fig. 1 for the ‘Preferred Reporting Items for Systematic reviews and Meta-Analyses’ (PRISMA) flow diagram.

Table 1 provides a summary of review characteristics. The 20 systematic reviews^7^, ^8^, ^9^, ^10^, ^11^, ^12^, ^13^, ^14^, ^15^^,^^31^, ^32^, ^33^, ^34^, ^35^, ^36^, ^37^, ^38^, ^39^, ^40^, ^41^ were comprised of 374 RCTs with n = 73,175 participants (intervention and control), aged 14–50 years old, on average, across the studies. Ninety-seven RCTs were from North America, 88 from Asia, 96 from Europe, 28 from Australia, 23 from the Middle East, 13 from South America, and one from Africa. Three reviews did not report study country. Six reviews focused on the prenatal period,^9^^,^^10^^,^^13^^,^^36^^,^^40^^,^^41^ five on the postpartum period^11^^,^^12^^,^^33^^,^^37^^,^^38^ and nine on the entire perinatal period.^7^^,^^8^^,^^14^^,^^15^^,^^31^^,^^32^^,^^34^^,^^35^^,^^39^ There was a range of interventions, with yoga being the most common (9 reviews).^8^, ^9^, ^10^^,^^12^^,^^13^^,^^32^^,^^36^^,^^40^^,^^41^ Comparisons included standard care, no intervention, waitlist or active controls (e.g., parenting education, social support). Outcomes were measured using self-report screening tools or diagnostic interviews (Supplementary Table S11). The majority of studies included in the reviews used the Edinburgh Postnatal Depression Scale (EPDS; 20 reviews) and Center for Epidemiologic Studies Depression Scale (CES-D; 15 reviews).

**Table 1:** Summary of review characteristics.

	Countries of the included studies	Number of RCTs	Sample size	Population (Age)	Time period	Intervention, PA intensity, PA type	Outcome	Outcome measurement tool
Carter et al. (2019)^11^	Australia (n = 3), UK (n = 3), USA (n = 6), Canada, Japan, Iran, Taiwan, India (each n = 1)	18	Primiparous or multiparous postnatal women (1428)	Range: majority 17–44 (based on 5 studies, 3 of which provided range and mean); Mean (I): 29.76 (13 studies); Mean (all): 28.3 (one study); one study provided no age data	postpartum	aerobic and/or strengthening and/or muscle stretching (n = 8), coaching and motivational health promotion techniques and no exercise (n = 4), mixed exercise and coaching/motivational promotion techniques (n = 6)	Postpartum depression symptoms; Secondary: Quality of life, anxiety	EPDS, CESD, HRSD; HRQoL IDAS-II
Daley et al. (2014)^10^	USA (n = 5), Columbia (n = 1)	6	Pregnant women (406)	14–38 years old	prenatal	aerobic exercise (n = 1), non-aerobic exercise (yoga) (n = 5)	Antenatal depression	CESD, EPDS
Davenport et al. (2018)^8^	USA (n = 8), Norway, UK, Spain (each n = 3), Australia, India (each n = 2), Brazil, Iran, Columbia, Japan (each n = 1)	26	Pregnant women without contraindication to exercise (7060)	Mean (I): 28.56 (23 studies); Mean (all):29.03 (3 studies)	prenatal	smart phone app PA intervention (n = 1); yoga (n = 8); counseling (n = 1); combined PA (n = 7); stationary cycling (n = 1); aerobic dance (n = 1); aerobic exercise (n = 2); stretching and breathing (n = 1); walking (n = 4)	Prenatal or postnatal depression diagnosis or symptoms OR Prenatal or postnatal anxiety diagnosis or symptoms	CESD, EPDS, health professional assessment
Gong et al. (2015)^13^	USA (n = 4), UK, India (each n = 1)	6	Pregnant women (375)	20–40 years old	prenatal	exercise-based yoga (n = 3), complex yoga interventions (n = 3)	Prenatal depression	CESD, POMS, EPDS, HADS
He et al. (2023)^14^	USA (n = 8) Spain (n = 6), China (n = 5), Australia, UK (each n = 4), Türkiye, Brazil, Egypt, Iran, India, Japan, Norway, Colombia (each n = 1)	35	Perinatal women (4935)	Mean (I): 29.64 (based on 23 studies); Mean (Con): 29.61 (based on 23 studies); Mean (all): 27.22 (based on 6 studies); Range: ≤29 & ≥30 (1 story), 17 to <40 (2 studies); one study provided no age data	prenatal or postpartum	water aerobics (n = 1); yoga (n = 8); general PA (n = 7); pram-walking (n = 3); combined (n = 7); aerobic (n = 2); stretching & breathing + education (n = 1); exercise + consultation (n = 1); group walking + support (n = 1); PA + consultation (n = 1)	Perinatal depression; perinatal depression severity	EPDS, CESD, HADS
Hicks et al. (2024)^31^	low to middle-income countries (n = 3), high-income countries (n = 16)	19	Women during pregnancy and the first year postpartum (23,589)	N/A	prenatal	general physical activity (n = 4); N/A (n = 7); moderate physical activity (n = 1); physical activity, including occupational and leisure (n = 1); aquatic (n = 1); moderate to vigorous (n = 2); yoga (n = 1); aerobic and strengthening (n = 1)	postpartum depression, postpartum anxiety, postpartum mental well-being, postpartum stress, postpartum distress, postpartum quality of life	EPDS, DASS, BDI, CESD, PRAQ, PGWBI, PSS, DABS, Structured Clinical Interview for DSM Disorders
Liu et al. (2022)^32^	USA (n = 5), Australia (n = 3), UK, China, Spain (each n = 2), Colombia, Norway, Egypt, Brazil, Turkey, Japan (each n = 1)	20	Pregnant and postpartum women (2866)	16 years and older	Prenatal or postpartum	physical exercise (n = 11), yoga (n = 3), walking (n = 2), exercise with babies, aerobic water exercise (each n = 1)	Prenatal or postnatal depression symptoms	EPDS, CESD, HDRS
McCurdy et al. (2017)^33^	North America, Europe, Asia, Middle East	16	Postpartum women (1327)	Range: 21–31 (based on 2 studies); Majority: 30 (based on one study); Mean (I): 29.72 (based on 12 studies), one study did not provide age data	Postpartum	Primarily aerobic exercise (n = 12), combined aerobic & resistance training (n = 2), flow yoga, whole-body gentle stretching (each n = 1). 3 studies also included a co-intervention (education, social support, dietary) that was not received by the control group	Postpartum depression symptoms	EPDS, CESD, HDRS, clinical interview
Morres et al. (2022)^34^	UK = 3, USA = 3, Spain = 2, Taiwan = 2, Australia = 1, Colombia = 1, Japan = 1 Iran = 1	14	Pregnant or postpartum women (2025)	Mean (I): 29.31	Prenatal or postpartum	aerobic exercise (pram walking, walking and various aerobic activites, dance, aerobic and strength training) (n = 11), PA + co-intervention (n = 3)	Perinatal depression symptoms	EPDS, CESD, BDI-II
Ji et al. (2024)^35^	Australia (n = 2), Spain (n = 5), China (n = 15), Turkey (n = 1), Brazil (n = 1), Iran (n = 3), UK (n = 3), India (n = 2), Egypt (n = 1), United States (n = 11), Japan (n = 1), Colombia (n = 1), Canada (n = 1)	48	Pregnant women (5282)	N/A	Prenatal	Aerobic exercise (n = 15), aerobic + resistance (n = 6), movement in water (n = 2), yoga (n = 18), fertility dance (n = 1), pelvic floor muscle training (n = 1), gymnastics (n = 2), stroller-walk (n = 2), Yoga + rehabilitation (n = 1)	Perinatal depression	BDI, CESD, SAS, HAMA, SDS, PHQ-9, HDRS, HADS, STAI, EPDS, SCL-90
Nakamura et al. (2019)^7^	USA (n = 6), Europe (n = 11), Canada (n = 1), Australia (n = 1), Iran (n = 1), South Korea (n = 1)	6	1163	Women during pregnancy and postpartum (25–32 for RCTs, unclear for observational studies)	Prenatal	combined types (n = 3), yoga (n = 1), stretching & breathing (n = 1), aerobic exercise (n = 1)	Postpartum depression	EPDS, CESD
Ng et al. (2019)^36^	USA (n = 4), UK (n = 1), India (n = 1)	6	405	Pregnant women (N/A)	prenatal	yoga-based intervention (n = 5)	Prenatal depression	EPDS, STAI, CESD, POMS, HADS, PANAS-N, QIDS
Pentland et al. (2022)^37^	Australia (n = 2), Canada (n = 1), UK (n = 2)	5	242	Postpartum women	Postpartum (mean: 28.9)	pram-walking (n = 1), pram-walking + social support (n = 1), aerobic and strength exercise (at least 50% pram-walking) (n = 1), consultation to promote exercise/pram-walking (n = 2)	Postpartum depression	EPDS
Pritchett et al. (2017)^38^	USA (n = 4), UK (n = 3), Australia (n = 2), Canada, India, Japan, Taiwan (each n = 1)	13	1734	Women <1 year postpartum (N/A)	Postpartum	Group exercise program + co-intervention (n = 2), Exercise counseling (n = 3), Exercise program (n = 1), Exercise counseling + dietary co-intervention (n = 2), Group exercise program (n = 12), Exercise counseling with group exercise (n = 1)	Postpartum depressive symptoms	EPDS, BDI, HAM-D3, DASS, CESD, PHQ-9, Structured clinicial interview (SCID)
Wang et al. (2023)^12^	Americas (n = 3), Oceania (n = 3), Europe (n = 3), Asia (n = 2), Africa (n = 1)	12	1260	Women with postpartum depression (Range: 20–35 (based on 2 studies), Mean (all): 29.31 (based on 10 studies))	Perinatal	pram walking (n = 4), supervised mixed exercise (n = 5), yoga (n = 3)	Postpartum depression	EPDS, HDRS
Xu et al. (2023)^15^	China (n = 6), India (n = 1), Iran (n = 2), America (n = 5), Australia (n = 3), Turkey (n = 1), UK (n = 4), Brazil (n = 1), Canada (n = 1), Spain (n = 1), Japan (n = 1)	26	2867	Pregnant or postpartum women Mean (I): 29.50 (based on 22 studies), N/A (based on 4 studies)	Postpartum	dance (n = 3), walking or combined (n = 7), yoga (n = 4), jogging (n = 1), body shape (n = 1), aerobic (n = 5), stretching (n = 2), exercise ball (n = 1), swimming combined (n = 1)	Postpartum depression	EPDS
Yuan et al. (2022)^39^	China, Norway, USA, Spain (each n = 2), Canada, Iran (each n = 3), UK, Brazil, Turkey, India, Switzerland (each n = 1)	14	4067	Pregnant women up to one year postpartum (Mean (all): 30.08 (based on 6 studies), Range: 16–50 (based on 6 studies), N/A (based on 2 studies)	Perinatal	aerobic (n = 1), stretching (n = 1), pilates and/or yoga (n = 3), combined (n = 8), stretching and breathing (n = 3), walk (n = 1), not specified (n = 1), exercise + household activities or leisure (n = 1)	Postpartum depression	EPDS, CESD, HDRS
Zhang et al. (2024)^9^	N/A	7	524	Pregnant women	Prenatal	high intensity interval training (n = 1), combined (n = 3), yoga (n = 3)	Prenatal depression	CESD, EPDS, HADS, POMS, BDI-II
Zhu et al. (2021)^40^	China (n = 7), USA (n = 8), UK (n = 2), India, Saudi Arabia, Spain, Colombia, Australia, Iran, and one from English speaking country (each n = 1)	24	1453	Pregnant women (N/A)	Prenatal	Yoga (n = 10), Massage (n = 4), Music (n = 6), Exercise (n = 4)	Prenatal depression	EPDS, CESD, HADS, DASS-21, PHQ9, SDS, HAMD, HAM-A, SAS, STAI
Miao et al. (2026)^41^	USA (n = 7), Taiwan (n = 2), Sweden (n = 3), China (n = 6), Hong Kong (n = 1), Nertherlands (n = 2), Iran (n = 9), Türkiye (n = 13), UK (n = 1), Germany (n = 2), Australia (n = 1), Japan (n = 1), Brazil (n = 1), India (n = 2), Spain (n = 1)	53	10,167	Pregnant women (29.86)	Prenatal	Yoga (n = 10), MBCP (n = 12), CBT (n = 11), Music (n = 15), MBSR (n = 6)	Prenatal depression, prenatal anxiety	EPDS, POMS

Abbreviations: I, Intervention; BDI/BDI-2, Beck Depression Inventory; CES-D, Center for Epidemiological Studies—Depression; DASS/DASS-21, Depression Anxiety Stress Scale; DABS, Derogatis Affects Balance Scale; EPDS, Edinburgh Postnatal Depression Scale; HRSD/HDRS/HDS, Hamilton Rating Scale for Depression; HAMD/HAM-D, Hamilton Scale; HADS, Hospital Anxiety and Depression Scale; HAM-A, Hamilton Anxiety Rating Scale; IDAS, Inventory of Depression and Anxiety Symptoms; PANAS, Positive and Negative Affect Schedule; PHQ-9, Patient Health Questionnaire; PSS, Perceived Stress Scale; PGWBI, Psychological General Well-being Index; PRAQ, Pregnancy-related Anxiety Questionnaire; POMS, Profile of Mood States; QIDS, Quick Inventory of Depressive Symptomology; STAI, State-Trait Anxiety Inventory; SDS, Zung Self-rating Depression Scale; SAS, Zung Self-rating Anxiety Scale; SCL-90, Symptom Checklist-90; N/A, Not available.

**Fig. 2:**
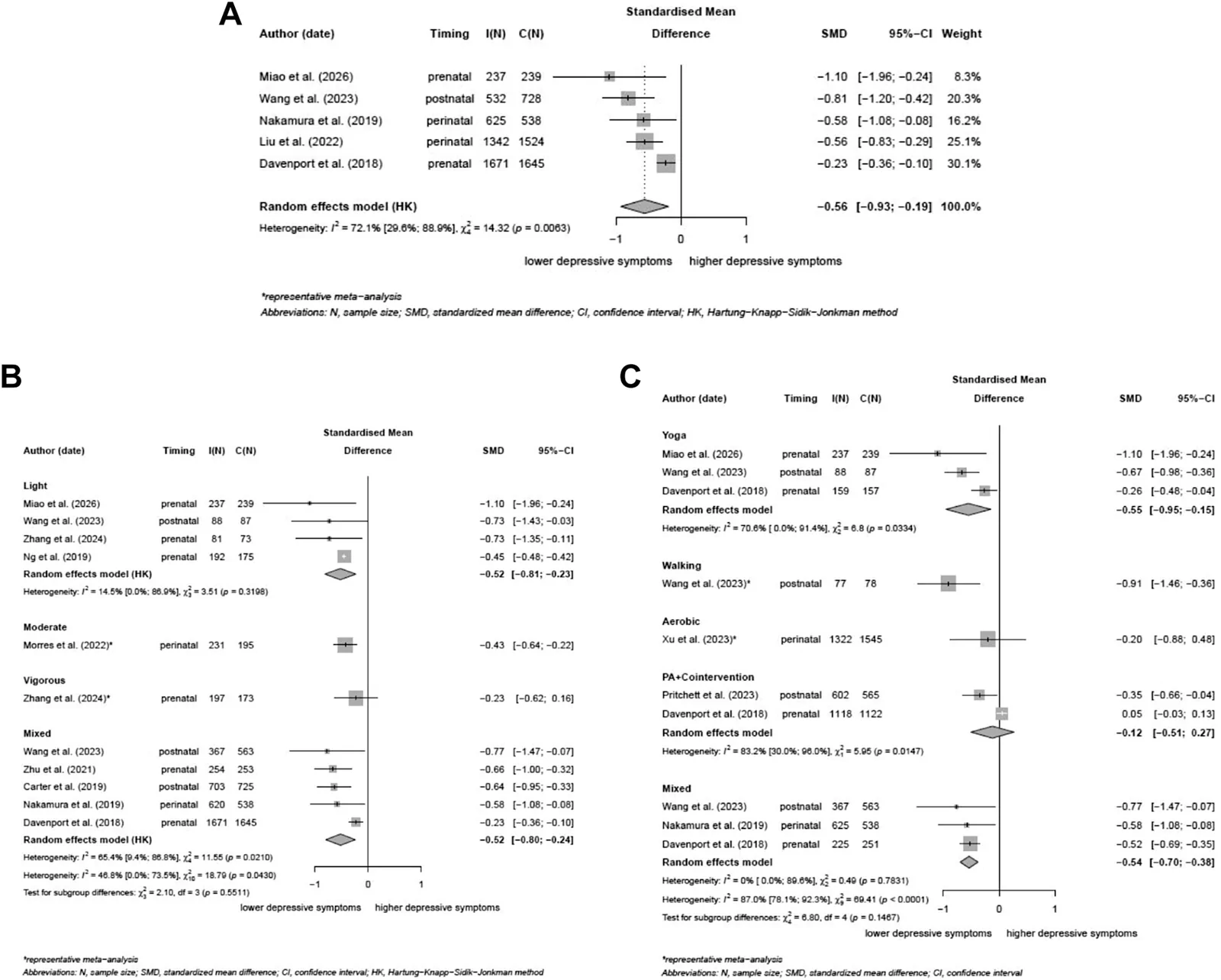
Meta-meta-analyses of the association between physical activity and depressive symptoms (A), physical activity intensity and depressive symptoms (B), and physical activity type and depressive symptoms (C).

For the main assessment, we conducted a meta-meta-analysis for overall PA and perinatal depressive symptoms. Of the 20 included meta-analyses, 16^7^, ^8^, ^9^, ^10^, ^11^, ^12^, ^13^^,^^15^^,^^31^, ^32^, ^33^, ^34^^,^^36^^,^^38^^,^^40^^,^^41^ could be included in the meta-meta-analysis. Four studies were removed due to unclear values in the synthesis^35^^,^^37^^,^^39^ and overuse of subgroups^14^ (Supplementary Table S12). After adjusting for CCA overlap,^28^ five systematic reviews^7^^,^^8^^,^^12^^,^^32^^,^^41^ were included in the meta-meta-analysis (CCA: 8.49%). The final analysis revealed that PA interventions were associated with a moderate reduction in perinatal depressive symptoms according to standard Cohen's d thresholds (SMD: −0.56; 95%-CI: −0.93 to −0.19). (Fig. 2A).

Due to high CCA overlap, we also summarized the results for overall PA narratively by subgroup. Seven reviews^8^, ^9^, ^10^^,^^13^^,^^36^^,^^40^^,^^41^ addressed prenatal effects of PA interventions on prenatal depressive symptoms, reporting a significant effect (SMD range: −0.23 to −1.10). Three reviews^7^^,^^8^^,^^31^ considered the effects of prenatal PA on postpartum depressive symptoms, however effect sizes were inconclusive (SMD: −0.58 [significant],^8^ 0.05 [not significant],^7^ one unclear^31^). Four reviews^11^^,^^12^^,^^33^^,^^38^ assessed postpartum PA and postpartum depressive symptoms: three showed significant results (SMD range: −0.34 to −0.81) while one review^33^ did not. Three reviews^15^^,^^32^^,^^34^ analyzed PA interventions spanning the entire perinatal period. Two showed significant effects (SMD: −0.21,^34^ −0.56^32^) while one effect^15^ was non-significant.

We conducted secondary assessments for PA intensity and PA types and perinatal depressive symptoms. For PA intensity, we used the Adult Compendium of PA^29^ to categorize the 16 eligible reviews^7^, ^8^, ^9^, ^10^, ^11^, ^12^, ^13^^,^^15^^,^^31^, ^32^, ^33^, ^34^^,^^36^^,^^38^^,^^40^^,^^41^ into light, moderate, vigorous, or mixed-intensity PA. Findings are reported in Supplementary Text S6. Meta-analyses were pooled for light and mixed-intensity PA; due to high overlap, the most recent, highest quality reviews were selected for moderate and vigorous intensities. The results showed that light (SMD: −0.52; 95% CI: −0.81 to −0.23), moderate (SMD: −0.43, 95%-CI: −0.64 to −0.22), and mixed-intensity (SMD: −0.52, 95%-CI: −0.80 to −0.24) PA were associated with a small-to-moderate, statistically significant decrease in depressive symptoms (Fig. 2B). Vigorous-intensity activity did not show a significant effect in the representative study (SMD: −0.23, 95%-CI: −0.62 to 0.16). Subgroup differences were not significant (χ^2^_3_: 2.10, p: 0.5511). Sensitivity analysis using the five reviews from the overall analysis confirmed these findings, though vigorous–intensity activity could not be included because the five reviews did not cover vigorous PA (Supplementary Figure S1).

Regarding the assessment of PA type, the included reviews encompassed a wide range of intervention types (e.g. yoga, dance, swimming), with yoga being the most common.^8^, ^9^, ^10^^,^^12^^,^^13^^,^^32^^,^^36^^,^^40^^,^^41^ Results are reported narratively in Supplementary Text S7. Meta-meta-analyses were conducted for yoga, mixed PA, and PA with co-interventions; representative reviews were selected for walking and aerobic activity (Fig. 2C). The meta-meta-analysis indicated that yoga (SMD: −0.55; 95%-CI: −0.95 to −0.15) and mixed PA types (SMD: −0.54, 95%-CI: −0.70 to −0.38) were associated with a moderate reduction in depressive symptoms. A robust meta-analysis for walking reported a strong effect on depressive symptoms (SMD: −0.91, 95%-CI: −1.46 to −0.36). Aerobic activity (SMD: −0.20, 95%-CI: −0.88 to 0.48) and PA with co-interventions (SMD: −0.12, 95%-CI: −0.51 to 0.27) showed no significant effect. Sensitivity analysis for yoga, walking, and mixed showed similar results (Supplementary Figure S2).

Further results on population, supervision and duration are reported in the supplement (Supplementary Text S8). A summary of the main findings for perinatal depressive symptoms can be found in Supplementary Table S13.

Five reviews assessed anxiety symptoms with three reporting effect sizes.^8^^,^^11^^,^^31^^,^^40^^,^^41^ Because two reviews assessed prenatal yoga and prenatal anxiety symptoms and had 0% overlap,^40^^,^^41^ we conducted a meta-meta-analysis, suggesting a strong reduction in anxiety symptoms for yoga (SMD: −0.85, 95%-CI: −1.29 to −0.42). A further review examining mixed PA interventions found no significant association for prenatal anxiety outcomes^8^ (SMD state anxiety: 0.06, 95%-CI −0.04 to 0.15; SMD trait anxiety: −0.21, 95%-CI −0.63 to 0.20). Pooled results were, therefore, also not significant (Supplementary Figure S3).

**Fig. 3:**
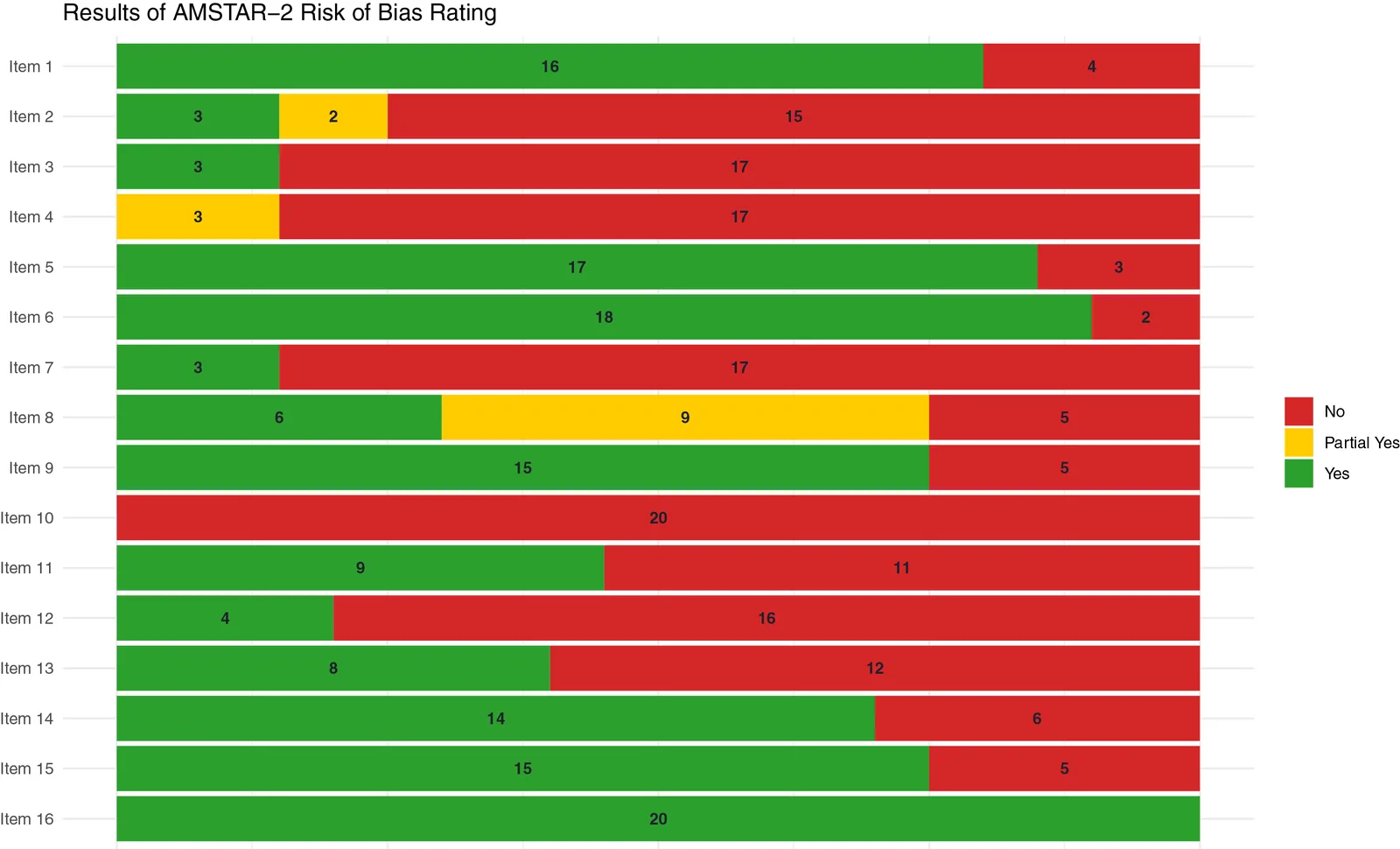
Results of AMSTAR-2 risk of bias rating for the 20 included systematic reviews.

Regarding risk of bias, twelve reviews^7^^,^^9^^,^^11^^,^^12^^,^^15^^,^^31^^,^^32^^,^^35^, ^36^, ^37^^,^^40^^,^^41^ assessed risk of bias in primary studies using the Cochrane ‘Risk Of Bias’ (ROB) tool (Version 1 or 2). Confidence in the results of the reviews was rated critically low using the AMSTAR-2 tool, largely due to gaps in reporting (Supplementary Table S8, Fig. 3). We used the GRADE approach to assess certainty of evidence for overall PA on perinatal depression symptoms and for yoga on prenatal anxiety symptoms; both were downgraded by one level, to moderate, due to the reviews' high risk of bias (Table 2). The other domains did not lead to a further downgrade of the certainty of evidence (Supplementary Table S14). Regarding reporting bias, the Egger's test indicated no evidence of bias across the full set of 16 reviews (p = 0.85; Supplementary Figure S4), and visual inspection of the funnel plots for perinatal depressive symptoms and prenatal anxiety also did not indicate bias (Table 2; Supplementary Figures S5–S8). However, these findings should be interpreted with caution, as neither method can fully rule out the presence of reporting bias.

**Table 2:** GRADE Certainty of evidence and summary of findings table.

Physical activity (PA) interventions compared to any non-PA comparison group (standard care, waitlist, active (non-PA) control, no care) for depressive symptoms in women during the perinatal period.
**Patient or population:** women in the perinatal period. **Setting:** not defined (various). **Intervention:** any type of physical activity intervention. **Comparison:** standard care, waitlist, active (non-PA) control, no care.

Outcomes	Relative effect (95% CI)	No of participants (studies)	Certainty of evidence (GRADE)	Comments
Perinatal depressive symptoms^b^	SMD^a^: −**0.56** (−0.93; −0.19)	5051 (16 systematic reviews with meta-analyses including 228 RCTs)	⊕⊕⊕⊖ Moderate^c^	Physical activity interventions may reduce perinatal depressive symptoms.
Prenatal anxiety symptoms^d^	SMD^a^: −**0.85** (−1.29; −0.42)	1105 (33 systematic reviews with meta-analyses including 87 RCTs)	⊕⊕⊕⊖ Moderate^c^	Yoga interventions may reduce prenatal anxiety symptoms.

**CI**, Confidence Interval; **SMD**, Standardized Mean Difference.

a Interpretation of SMD effect sizes: 0.2–0.5 = small effect sizes; 0.5–0.8 = medium effective sizes; >0.8 = large effect sizes.

b Outcome: Perinatal depressive symptoms assessed with EPDS, HDRS, STAI, PANAS-N, CESD, POMS, HADS, QIDS, or by health care provider.

c Evidence downgraded 1 level due to concerns about high risk of bias in the included reviews.

d Outcome: Perinatal anxiety symptoms assessed with S-STAI, STAI, SAS, HAMA, HADS, POMS, EPDS.

## Discussion

This umbrella review with meta-meta-analysis synthesized global evidence from 20 systematic reviews including 374 RCTs and 73,175 participants on the effects of PA on perinatal depressive symptoms and anxiety symptoms. Our meta-meta-analysis provides high-level moderate-certainty evidence that PA interventions are likely associated with a reduction in depressive symptoms among pregnant and postpartum women and demonstrates—for the first time—consistent effects across various intensities (light, moderate, and mixed) and PA types, such as yoga and mixed activities. Evidence for anxiety symptoms was limited, but effect estimates for yoga and prenatal anxiety indicate potential strong benefits.

The WHO^17^ and national networks recommend at least 150 min of moderate-intensity aerobic PA per week during pregnancy and postpartum, unless medically contraindicated, while light activity (e.g., easy walking or cycling) is only noted as a replacement for sedentary time. Our findings suggest that even light-intensity activity can improve perinatal depressive symptoms, aligning with recent evidence^42^ that shows associations between light-intensity PA and a lower risk of mortality in the general population. This new evidence across population groups underscores the significant potential of light activity, which includes many feasible forms, such as yoga or walking, to improve health. PA guidelines should explicitly promote light activities alongside moderate-intensity activities for mental health. Encouraging light activity may support habit formation and, over time, help build towards regular moderate-intensity exercise, especially among those who are not regularly active.

Pooled effect sizes for yoga and mixed activity interventions demonstrated small-to-moderate and statistically significant reductions in depressive symptoms, supporting prior evidence suggesting that mind-body practices are particularly beneficial during the perinatal period.^43^ Notably, mixed activity interventions showed an even higher effect size than yoga, potentially due to their broader physiological impact, combining endurance, strength, and flexibility components.

Previous systematic reviews in the general population have found that moderate to high-intensity PA (defined as MET-minutes/week) is associated with lower levels of anxiety.^44^ However, our findings could not fully confirm this in pregnant and postpartum women. These discrepancies may reflect variability in PA type, intensity, timing, and anxiety measures across studies. Overall, fewer studies examined anxiety than depression, and those that did often included anxiety as a secondary outcome. Consequently, interventions may not have targeted anxiety specifically, potentially reducing the efficacy of these interventions. Mind-body approaches such as yoga may be especially beneficial, which may explain clearer effects for yoga-based interventions compared to other PA types, as was the case in our study.^40^^,^^41^ Despite inconsistencies, PA could help reduce perinatal anxiety symptoms; however, further research is needed.

Despite international recommendations supporting PA for perinatal depression and anxiety,^17^ many clinical practice^45^ and public health^46^ guidelines provide limited concrete recommendations. Moving forward, the focus must shift from evidence generation to implementation and emphasize a multi-step, multi-level approach focusing particularly on the contextual factors that promote effective and long-term PA practices.^47^ Raising awareness among health care professionals and perinatal women regarding the benefits of PA for depression and anxiety–including debunking common myths that PA is unsafe during pregnancy or postpartum–is a crucial first step. Beyond mental health, new evidence points to further benefits of PA on physical health during the perinatal period, including for cardiometabolic health,^48^ further underlining its importance in perinatal health care. Training and educational programs for providers focused on building the knowledge and confidence needed to promote perinatal PA^49^ should be expanded across health care, community and social care settings, and varying global contexts could support PA promotion along perinatal care pathways.

Beyond the health system, widespread campaigns are essential to highlight the importance of PA for perinatal depression and anxiety. Current initiatives mostly target the general population, but more should engage directly with pregnant and postpartum women. This would raise awareness in perinatal populations and encourage key stakeholders to invest in supportive environments. Contextual factors, including income, physical and social environments, pre-pregnancy PA status, and race and ethnicity, must be considered in intervention design and implementation, and perinatal women must be involved in co-designing programs that meet their needs. In low- and middle-income countries, where infrastructure and resources are often limited, focusing on primary care, multicomponent interventions, and non-specialist delivery may help promote sustainable programs.^50^ Digital interventions may also offer flexible, accessible ways to engage in PA across different settings.

The key strength of this study is its unique, integrated perspective, broadening the generalizability of findings beyond single meta-analyses and offering practical guidance for clinical, public health, and policy contexts. We worked with the highest level of evidence, including only RCTs, a global perspective, and meta-meta-analysis. Given limited guidance for umbrella reviews with meta-meta-analysis, we adapted some approaches (e.g., meta-analysis of few meta-analyses, GRADE) from Cochrane systematic review guidelines. This represents both a strength and a potential limitation, highlighting the need for clearer methodological guidance. Another limitation is that umbrella reviews can only synthesize already published findings; therefore, we were not able to draw clear conclusions for anxiety symptoms or clinical significance. The inability to perform meta-meta-analysis for anxiety symptoms beyond prenatal yoga constitutes a limitation, as conclusions rely mostly on narrative synthesis, highlighting the need for more high-quality research on PA and perinatal anxiety. In addition, the strong effect for prenatal yoga and anxiety symptoms should be interpreted with caution because available data remains limited and a further review suggested non-significant results for mixed PA and prenatal anxiety symptoms.^8^ Heterogeneity, potentially due to varying intervention and outcome measures, may have affected our review and synthesis. Varying levels of reporting in systematic reviews and primary studies may have influenced the critically low rating by the AMSTAR-2 tool, meaning we had to downgrade for risk of bias in GRADE, but not for any other domain. Although we aimed to minimize overlap using the CCA, residual overlap remains a limitation. This may cause some primary studies to be weighted more greatly in the study and may reduce the assumption of independence between meta-analyses. Where pooling was not possible, we selected representative meta-analyses, which may have resulted in some information loss. The representative study for walking showed a high effect with wide CIs, which could not be confirmed by further analyses and should therefore be interpreted with caution due to potential small-study-effects or bias. We aimed to mitigate bias in this approach by only selecting recent, high-quality meta-analyses. Subgroup difference tests for PA intensity should also be interpreted cautiously given the small number of studies per subgroup. Although we aimed to provide a global perspective, low- and middle-income countries were underrepresented, which should be considered in applying these findings to those settings.

This study synthesized available global evidence on the overall as well as intensity- and type-specific benefits of PA for perinatal depressive symptoms. Our findings call for the inclusion of light, moderate, or mixed-intensity PA as feasible public health interventions to improve perinatal depression and, potentially, anxiety. Next steps should focus on translating this evidence into practice. This includes more specificity around PA intensities and types in global and national guidelines as well as ensuring that PA is integrated into perinatal care pathways as a key component of protecting and promoting the mental health of pregnant and postpartum women.

## Contributors

EM, HLW, DP, and MB conceptualized the review and planned the methodological approach and analysis. EM conducted the literature search and performed screening with HLW and data extraction and risk of bias assessments with MK and AP. EM and HLW conducted the formal statistical analysis and drafted tables and figures. All authors were involved in interpreting the data. EM, HLW, RG, and MB drafted the initial manuscript and all authors were involved in further reviewing and finalizing of the manuscript. EM and HLW accessed and verified underlying data reported in the manuscript. All authors had full access to all the data and had final responsibility for the decision to submit for publication.

## Data sharing statement

The data used in this study is available in the article or in the Appendix.

## Declaration of interests

Prof. Dr. Bujard reports receiving payment or honoraria for lectures, presentations, or educational events from the Institute for Quality and Efficiency in Health Care and Local Alliances for Families. Prof. Dr. Bujard also reports participation on an advisory board for the Robert Koch Institute, Germany's Public Health institute.

All authors declare no competing interests.

## Appendix A Supplementary data

Supplement

Translated Abstract
