# Supplementary material for: Physical activity and perinatal depression and anxiety: a global umbrella review and meta-meta-analysis
Source: eClinicalMedicine. 2026 Jul 19;98:104054. doi: 10.1016/j.eclinm.2026.104054 (PMC13523840; doi:10.1016/j.eclinm.2026.104054)
Supplement: Supplementary file 1 [file mmc1.docx]

**Appendix**

Table of Contents

[Supplementary text	2](#_Toc229645951)

[Supplementary text 1: Systematic review criteria	2](#_Toc229645952)

[Supplementary text 2: AMSTAR-2 Guidance Document	2](#_Toc229645953)

[Supplementary text 3: Rationale for meta-meta-analysis	6](#_Toc229645954)

[Supplementary text 4: Corrected covered area: formula and interpretation	7](#_Toc229645955)

[Supplementary text 5: Applying GRADE to Umbrella Reviews	7](#_Toc229645956)

[Supplementary text 6: Narrative review of physical activity intensity and perinatal depressive symptoms	7](#_Toc229645957)

[Supplementary text 7: Narrative review of physical activity type and perinatal depressive symptoms	8](#_Toc229645958)

[Supplementary text 8: Further results on population, supervision, and duration	8](#_Toc229645959)

[Supplementary figures	9](#_Toc229645960)

[Supplementary figure 1: Sensitivity analysis: Physical activity intensity and perinatal depressive symptoms	9](#_Toc229645961)

[Supplementary figure 2: Sensitivity analysis: Physical activity type and perinatal depressive symptoms	9](#_Toc229645962)

[Supplementary figure 3: Meta-meta-analysis: PA and prenatal anxiety symptoms	10](#_Toc229645963)

[Supplementary figure 4: Egger’s test: Review pool	10](#_Toc229645964)

[Supplementary figure 5: Funnel plot: physical activity and perinatal depressive symptoms	11](#_Toc229645965)

[Supplementary figure 6: Funnel plot: physical activity intensity and perinatal depressive symptoms	12](#_Toc229645966)

[Supplementary figure 7: Funnel plot: physical activity type and perinatal depressive symptoms	12](#_Toc229645967)

[Supplementary figure 8: Funnel plot: physical activity and prenatal anxiety symptoms	13](#_Toc229645968)

[Supplementary tables	13](#_Toc229645969)

[Supplementary table 1: PICO Table	13](#_Toc229645970)

[Supplementary table 2: Search strategy	13](#_Toc229645971)

[Supplementary table 3: Reasons for exclusion of studies from the systematic literature search, after screening for title and abstract	18](#_Toc229645972)

[Supplementary table 4: PRIOR checklist for overviews of reviews (Gates et al., 2022)	19](#_Toc229645973)

[Supplementary table 5: Fusar-Poli and Radua’s (2018) ‘Ten simple rules for conducting umbrella reviews’	20](#_Toc229645974)

[Supplementary table 6: Deviations from the protocol	20](#_Toc229645975)

[Supplementary table 7: Detailed AMSTAR-2 risk of bias assessment	21](#_Toc229645976)

[Supplementary table 8: Overview of studies for meta-meta-analysis and overlap adjustment	24](#_Toc229645977)

[Supplementary table 9: Physical activity intensity MET value cut-offs	24](#_Toc229645978)

[Supplementary table 10: Reporting of GRADE assessments in the included systematic reviews	25](#_Toc229645979)

[Supplementary table 11: Perinatal mental health outcome measurement tools	26](#_Toc229645980)

[Supplementary table 12: Reasons for exclusion of meta-analyses	30](#_Toc229645981)

[Supplementary table 13: Summary of main findings for perinatal depressive symptoms	31](#_Toc229645982)

[Supplementary table 14: GRADE Evidence Profile	32](#_Toc229645983)

[References	33](#_Toc229645984)

# **Supplementary text**

## **Supplementary text S1: Systematic review criteria**

Systematic review criteria derived from the Cochrane handbook:

- Clear research question (using PICO)
- Comprehensive and explicit search (keywords & MESH terms provided, minimum of two databases screened)
- Clearly defined inclusion and exclusion criteria
- Quality appraisal/RoB assessment

And: Formal synthesis (in our case only with meta-analysis)

## **Supplementary text S2: AMSTAR-2 Guidance Document**

Source: Shea BJ, Reeves BC, Wells G, Thuku M, Hamel C, Moran J, Moher D, Tugwell P, Welch V, Kristjansson E, Henry DA. AMSTAR 2: a critical appraisal tool for systematic reviews that include randomised or non-randomised studies of healthcare interventions, or both. BMJ. 2017 Sep 21;358:j4008.

Many of the items in AMSTAR 2 are written to be self-explanatory. However, the underlying issues are often complex, and subject to varying interpretation, particularly when judgments are made across a wide spectrum of interventions. Here we provide additional guidance on use of AMSTAR 2. Material in this document overlaps with that in the published paper. This is intentional, as this Appendix is intended to be a stand-alone document. We emphasise this is guidance – it gives an indication of how we think the criteria should be applied in settings where reviews are conducted of well-defined (usually clinical) interventions. Individual users, of course, may find it necessary to deviate from the guidance both in addressing individual domains and in making an overall appraisal of a systematic review. We ask that in doing so they document these variations so that others can benefit from their experiences AMSTAR 2 is not designed to generate an overall ‘score’. A high score may disguise critical weaknesses in specific domains, such as an inadequate literature search or are a failure to assess risk of bias (ROB) with individual studies that were included in a systematic review. In making an overall rating of systematic review it is important to take account of flaws in critical domains, which may greatly weaken the confidence that can be placed in a systematic review.

Item 1: Did the research questions and inclusion criteria for the review include the components of PICO?
It is common practice to use PICO description (population, intervention, control group and outcome) as an organising framework for a study question. Sometimes timeframe should be added if this is critical in determining the likelihood of a study capturing relevant clinical outcomes (e.g. an effect of the intervention is only expected after several years). PICO identifies the elements that should be described in detail in the report of the systematic review and should enable the appraiser to judge selection of studies, and their combinability, and enable the user of the review to determine applicability of the results. Authors of systematic reviews do not always make the elements of PICO explicit but they should be discernable through a careful reading of the abstract, introduction and methods sections. To score ‘Yes’ appraisers should be confident that the 4 elements of PICO are described somewhere in the report.

Item 2: Did the report of the review contain an explicit statement that the review methods were established prior to conduct of the review and did the report justify any significant deviations from the protocol?
Systematic reviews are a form of observational research and the methods for the review should be agreed on before the review commences. Adherence to a well-developed protocol reduces the risk of bias in the review. Authors should demonstrate that they worked with a written protocol with independent verification. This can take the form of registration (e.g. at PROSPERO - https://www.crd.york.ac.uk/PROSPERO/ ), an open publication journal (e.g. BMJ Open) or a dated submission to a research office or research ethics board. The research questions and the review study methods should have been planned ahead of conducting the review. At a minimum this should be stated in the report (scores ‘Partial Yes’). To score ‘Yes’ authors should demonstrate that they worked with a written protocol with independent verification (by a registry 2 | P a g e or another independent body, e.g. research ethics board or research office) before the review was undertaken. Appraisers should compare the published report of the review with the registered protocol, when the latter is available. If there are deviations from the protocol appraisers should determine whether these are reported and justified by review authors. Obvious unexplained discrepancies should result in downgrading of the rating.

Item 3: Did the review authors explain their selection of the study designs for inclusion in the review? The selection of study types for inclusion in systematic reviews should not be arbitrary. The authors should indicate that they followed a strategy. The general rule (this may have to be inferred from what the authors actually wrote) is that they asked first whether a review restricted to RCTs would have given an incomplete summary of the effects of a treatment. This might be because there were no relevant RCTs or because of missing outcomes in available RCTs [usually harms], inadequate statistical power, restrictive populations, or unrepresentative control/intervention treatments. If the answer to this general question is yes the inclusion of nonrandomized studies of the intervention(s) is justified. Conversely, to justify restriction of the review to RCTs the authors should argue that they can provide a complete picture of the effects they are interested in. Restriction of a review to only NRSI is justified when RCTs cannot provide the necessary outcome data, or in the case where reviews of RCTs have been completed and the review of NRSI will complement what is already known. Inclusion of both RCTs and NRSI may be justified to get a complete picture of the effectiveness and harms associated with an intervention. In this situation we recommend (see below) that these two types of studies are assessed and combined independently (if meta-analysis is appropriate). This is a somewhat neglected area and even with guidance it can be difficult to judge the extent to which a review meets the rating criteria. The justification for selection of study designs may have to be inferred from a careful reading of the complete study report.

Item 4: Did the review authors use a comprehensive literature search strategy?
At least two bibliographic databases should be searched. The report should include years and databases examined (e.g., Central, EMBASE, and MEDLINE). Key words and/or MESH terms should be reported and the full search strategy available on request. Searches should be supplemented by checking published reviews, specialized registers, or experts in the particular field of study, and by reviewing the reference list from the studies found. Sometimes it is necessary to approach authors of original studies to clarify results or obtain updates or corrections. Publications in all relevant languages should be sought and a justification provided when there are language restrictions. We have highlighted the need for searching the grey literature in some cases. Grey literature is sometimes important with reports of policy and program evaluations that are only available from web sites (e.g. government, non-government or health technology agencies). These may or may not have been subject to peer review and such appraisals should be looked for. Where the grey literature is considered important, authors should have searched appropriate sources, such as trial registries, conference abstracts, dissertations, and unpublished reports on personal websites (e.g. universities, ResearchGate). In addition, trials of medical interventions may not have been published in peer-reviewed journals but can be obtained directly from company sponsors or directly from investigators. To score ‘Yes’ appraisers should be satisfied that all relevant aspects of the search have been addressed by review authors.

Item 5: Did the review authors perform study selection in duplicate?
Best practice requires two review authors to determine eligibility of studies for inclusion in systematic reviews. This involves checking the characteristics of a study (from title, abstract and full text) against the elements of the research question In the response options, we point to the desirability of review authors describing inter-rater agreement across a sample of studies being considered for inclusion in the review. A consensus process should have been used when disagreements arose in study selection. If one individual carried out selection of all studies, with a second reviewer checking agreement on a sample of studies, we recommend that a Kappa score indicating ‘strong’ agreement (0.80 or greater) should have been achieved. There should have been at least two independent assessors for study selection. A consensus process should have been used when disagreements arose in study selection. In the event that one individual carried out selection of studies a second reviewer should have checked agreement on a sample of representative studies and they should have achieved a kappa score of 0.80 or greater.

Item 6: Did the review authors perform data extraction in duplicate?
As in Item 5, there should have been at least two independent assessors performing data extraction. A consensus process should have been used when disagreements arose. In the event that one individual carried out data extraction a second reviewer should have checked agreement on a sample of studies and they should have achieved a kappa score of 0.80 or greater.

Item 7: Did the review authors provide a list of excluded studies and justify the exclusions?
This item requires review authors to provide a complete list of potentially relevant studies with justification for the exclusion of each. Non-inclusion of studies may be necessary for a range of reasons, based on inappropriate/ irrelevant populations, interventions and controls. Exclusion should not be based on risk of bias, which is dealt with separately and later in the review process. Unjustified exclusion may bias the review findings and we encourage an inclusive approach in the early stages of a review. This item requires review authors to provide a complete list of potentially relevant studies with justification for the exclusion of each one.

Item 8: Did the review authors describe the included studies in adequate detail?
The description of subjects, interventions, controls, outcomes, design, analysis and settings of the studies should be provided. The detail should be sufficient for an appraiser, or user, to make judgments about the extent to which the studies were appropriately chosen (in relation to the PICO structure) and whether the study populations and interventions were relevant their own practice or policy. The descriptors also provide a framework for studying heterogeneity in intervention effects (e.g. by dose, age range, clinical setting etc.)

Item 9: Did the review authors use a satisfactory technique for assessing the risk of bias (RoB) in individual studies that were included in the review?
This is a crucial part of the appraisal of any systematic review, particularly those that include non-randomized studies of interventions (NRSI). The key appraisal question is whether review authors have taken account of the risk of bias when summarising and interpreting the results. When the review is confined to randomized controlled trials (RCTs) we recommend that you consult the Cochrane Handbook to determine whether the review authors have made an adequate assessment of ROB with individual RCTs. This section is concerned with the challenge posed by RoB in non-randomised studies. Review authors should have used a systematic approach to ROB assessment, preferably with a properly developed rating instrument. If they have used a non-standard instrument you should be satisfied that it was capable of detecting serious methodological flaws. Several ROB instruments (for individual studies) are in common use, including the Newcastle Ottawa Scale, SIGN, and the Mixed Methods Appraisal Tool (MMAT). The most comprehensive assessment instrument is the recently introduced Cochrane instrument, ROBINS-I. It is appreciated that this instrument may not have been available at the time a review was performed. In developing AMSTAR 2 we drew on the Cochrane RoB instruments for RCTs: (http://handbook.cochrane.org/chapter_8/8_5_the_cochrane_collaborations_tool_for_assessing_r isk_of_bias.htm) and NRSI: www.riskofbias.info. In both cases the domain appraisal items are drawn from these instruments. Whatever instrument was used by the review authors, appraisers should be satisfied that it addresses the items listed in item 9 of the instrument. Please note that the guidance given here is not comprehensive – AMSTAR 2 addresses only the most commonly recognised domains of bias. A deeper assessment of risk of bias requires specialist input. In assessing how RoB has been assessed by review authors you should seek methods and content expert advice, if that is not included in your team. Advisors should be asked to provide specific advice on which confounders are important, how to identify selection and measurement biases that are likely, to be relevant to the review under consideration. In addition, you should seek guidance on what adjustment techniques for confounding would be appropriate. The following list of domains of bias has been selected from the ROBINS-I/ACROBAT-NRSI instrument as being the most relevant to systematic reviews that include NRSI: Confounding. Confounding occurs when the effects of two associated interventions or exposures (e.g. smoking and alcohol consumption) have not been separated during analysis. This can result in an effect being attributed to one variable when it is due to the other. In the study of interventions confounding may also be related to the indication for treatment, for instance when one drug is given preferentially to patients with higher rates of co-morbidities than the comparator drug and where these co-morbid conditions are associated with the outcome of interest. These assessments are typically quantified in the baseline data reported in the individual study. Potential baseline confounding can be addressed in several ways, including design (eg matching by propensity score), adjustment (e.g. logistic regression) and other techniques such as instrumental variable analysis and the inclusion of ‘tracer’ exposures. It is common to assume that even sophisticated techniques will not adjust completely for all confounders, meaning weak associations, even if statistically significant, should be interpreted cautiously. Sample selection bias. This occurs when subjects are sampled in a biased way that directly distorts the true relationship between exposure and outcome. It requires no third 5 | P a g e factor, as is the case with confounding. For instance, you should not study the association between smoking and heart disease by recruiting subjects referred to a smoking cessation clinic. The selection of subjects with the exposure of interest should be unrelated to their outcome. Likewise, selection of subjects with the outcome should be unrelated to their exposure status. The timing of selection can be important. If subjects have been using a drug for some time before enrolment (prevalent users) they will be a tolerant group with a lower risk of adverse outcomes. For this reason, contemporary pharmaco-epidemiological studies recruit ‘new users’ of medications (analogous to starting treatment in a RCT). Other temporal sampling biases (immortal time bias and inception bias) are sometimes important. It is recommended that users refer to ROBINS-I guidance document for more information. Bias in measurement of exposures and outcomes: measurement of an exposure or treatment may be misclassified if there is no accurate recording made in real time. Typically, modern pharmaco-epidemiological studies use prescriptions or dispensing records as a surrogate for consumption. But adherence to dispensed treatment will not be 100% so actual consumption will be miss-classified by this method. If this error is nondifferential it will be a bias to the null. However, in some fields of research investigators rely on recall (e.g. ultraviolet exposure and melanoma). This may lead to differential misclassification. For instance, parents of a child who has died of SIDS may have heightened recall of any medications they administered to the baby prior to the event. Measurement of outcomes can also be affected by misclassification and if this is nondifferential it will usually be a bias to the null. However, non-differential misclassification can introduce bias. For instance, if leg ultrasound is performed frequently in women with swollen painful legs who are taking an oral contraceptive, selection of individuals from an ultrasound clinic may bias studies of the association between DVT and oral contraceptives. Selective reporting of outcomes and analyses: large observational studies may analyse population databases that record many outcomes occurring in a defined population. If outcomes are not pre-specified (preferably in a registered protocol) investigators may be tempted to analyse multiple outcomes and selectively report those that appear to be different between exposed and non-exposed individuals. In addition, there are usually several potential methods for analysing a non-randomized dataset (including, for example, different ways of categorising the intervention, or different multi-variable adjustment models). If the analytical protocol is not specified in advance of the study it may be possible to select one set of analyses that appears to show a significant statistical difference that is not apparent in the other analyses. Reviewers should determine whether study authors pre-specified outcomes and analyses. This will become easier as more studies are registered before being conducted.

Item 10: Did the review authors report on the sources of funding for the studies included in the review?
Several investigations have shown that commercially sponsored studies are more likely to have findings that favour a sponsor’s product than independently funded studies. It is valuable for 6 | P a g e review authors to document the funding sources for each study included in the review or to record that the information was not provided in the study reports. Depending on this information it may be possible to analyse separately the results from commercially funded and independently funded studies.

Item 11: If meta-analysis was justified did the review authors use appropriate methods for statistical combination of results?
(Only complete this item if meta-analysis of other data synthesis techniques were reported) Review authors should have stated explicitly in the review protocol the principles on which they based their decision to perform meta-analysis of data from the included studies. These include the desire to obtain a single pooled effect (for instance from a number of compatible but underpowered studies) and the extent to which the studies are compatible (in terms of populations controls and interventions) and therefore capable of being combined. Where meta-analysis was considered appropriate authors should have explained their decisions to use fixed or random effects models in the case of RCTs, and set out the methods they intended to use to investigate heterogeneity. With NRSI study populations vary greatly in size from small cohorts (of tens or hundreds of participants) to studies of hundreds of thousands of individuals and thousands of events. If these results, are going to be combined with those from smaller RCTs the pooled estimates of effect will be dominated by the data from the non-randomized studies. In addition, the results from NRSI may be affected by a range of biases (see above), meaning that the overall pooled estimates may be precise but biased. Review authors should report pooled estimates separately for the different study types. In the case of NRSI, pooling may result in a very precise and ‘statistically significant’, but biased, estimate of effect. However, the confidence interval is calculated on the assumption that there is no bias (i.e. the estimates are as accurate as if obtained from a high quality RCT with the same number of participants). It is rare for a NRSI to have as low risk of bias as a high quality RCT of the same research question and confidence intervals for NRSI (and pooled estimates based on NRSI) should be viewed with caution. This issue is important when considering the varying risk of bias, and uncertainty about the risk of bias across NRSI. Heterogeneity is an important issue in any meta-analysis. It is particularly important in a review of NRSI because of the more diverse methods that are likely to have been used across different studies. In addition to the usual sources of heterogeneity [different comparators, variations in baseline risk of outcomes or other characteristics of the study population, differing interventions (e.g. dose effects, context/setting, practitioner experience) and different definitions of outcomes], it is important to consider heterogeneity in source of participants, completeness of data, methods of data management and analysis. Statistical adjustment of intervention effects for confounders may result in estimates that are quite different from the unadjusted estimate derived from the raw data. Generally, when combining the results of NRSI review authors should pool the fully adjusted estimates of effect, not the raw data. If they do the latter there should be a clear justification. However, different studies are very likely to report treatment effects that have been adjusted for different sets of covariates (or covariates measured or fitted in different ways); this diversity represents another source of potential heterogeneity.

Item 12: If meta-analysis was performed did the review authors assess the potential impact of RoB in individual studies on the results of the meta-analysis or other evidence synthesis?
In cases where review authors have chosen to include only high quality RCTs there may be little discussion of the potential impact of bias on the results. But where they have included RCTs of variable quality they should assess the impact of this by regression analysis, or by estimating pooled effect sizes with only studies at low ROB. In the case of NRSI they should estimate pooled effect sizes while including only studies at low or moderate risk of bias, and/or only those at low ROB (if there are any). If meta-analyses (or other data synthesis techniques such as regression analysis) were not performed the authors should still provide some commentary on the likely impact of ROB on individual study results.

Item 13: Did the review authors account for RoB in individual studies when interpreting/ discussing the results of the review?
Even if meta-analyses were not conducted review authors should include discussion of the impact of ROB in the interpretation of the results of the review. This is always important, but especially when reviews include RCTs with variable ROB, and with any review that includes NRSI. This discussion should not be limited to the impact of ROB on the pooled estimates (see above), but should also consider whether it may account for differences between the results of individual studies. The authors should make an explicit consideration of ROB if they make any recommendations that are likely to have an impact on clinical care or policy.

Item 14: Did the review authors provide a satisfactory explanation for, and discussion of, any heterogeneity observed in the results of the review?
There are many potential causes of heterogeneity in the results of NRSI than in RCTs. Many factors considered in this instrument, including different study designs, different methods of analysis, different populations and differing intensities of the intervention(s) – dosages in the case of drugs. Both the PICO elements and the domains of bias listed in Item 9 should also be considered as important potential sources of heterogeneity in the results. Review authors should explore these possibilities and discuss the impact of heterogeneity on the results conclusions and any recommendations

Item 15: If they performed quantitative synthesis did the review authors carry out an adequate investigation of publication bias (small study bias) and discuss its likely impact on the results of the review?
This is a very important issue, but can be difficult for review authors and appraisers to resolve completely. Typically, statistical tests or graphical displays are used and if they are positive then it indicates the presence of PB. However, negative tests are not a guarantee of the absence of PB as the tests are insensitive. To some extent the importance of PB depends on context and setting. For instance, a series of apparently methodologically sound industry-sponsored studies (e.g. 8 | P a g e drugs, devices, putative toxins) might be more likely to be affected by PB than similar studies conducted independently of industry. The key issues are whether the authors have done their best to identify PB through deeper and intensive literature searches (as needed and according to the setting), shown an awareness of the likely impact of PB in their interpretation and discussion of the results and performed a sensitivity analyses to determine how many missing ‘null’ studies would be needed to invalidate the results they obtained.

Item 16: Did the review authors report any potential sources of conflict of interest, including any funding they received for conducting the review?
As noted above (under ROB), individual studies funded by vested interests may generate results that are more likely to favour the intervention than do independent studies. The same assumption applies to systematic reviews and authors should report their direct funding sources. Journals generally will require this. But assessment of the reviewers’ conflicts of interest doesn’t stop there. They should report their other ties. The review may be independently funded, but the authors have ties to companies that manufacture products included in the systematic review. Professional conflicts of interest are powerful, but harder to discern as they are seldom reported. When investigators have a career-long investment in a field of research, a review that conflicts with their long-held beliefs can be confronting. Potential conflicts of interest of this type will be hard to assess, but may be inferred from the fact that the reviewers have published extensively in the field being reviewed and their studies are included in the systematic review. While it can be argued that the effects of competing interests might manifest as flaws in the other domains of bias we believe that this item should always be rated separately.

## **Supplementary text S3: Rationale for meta-meta-analysis**

An umbrella review is necessary if, for instance, there are contrasting findings which may hinder the advancement of clinical knowledge, which we argue is the case in the context of physical activity interventions and perinatal depression and anxiety^2^. In a similar vein, Cochrane guidance for umbrella reviews^3^ states that re-analyzing outcome data may make sense if 1) the objective of the review is to answer a different clinical question, 2) if, most but not all, of the systematic reviews have analyzed specific populations and subgroups, 3) if different summary measures or models were used across the systematic reviews, 4) if the data included in the systematic review were not specifically analyzed in a meta-analysis. While Fusar-Polis & Radua^2^ and Pollock et al.^3^ do not explicitly address meta-meta-analysis, we argue that calculating an overall effect estimate for physical activity and perinatal depressive symptoms is necessary and valid due to the contrasting effect estimates across systematic reviews. In the case of PA type and PA intensity, not all systematic reviews have addressed these subgroups, suggesting that a meta-meta-analysis would be beneficial to understand the effects and provide clear evidence that can be applied in a clinical setting.

As part of the umbrella review approach, meta-meta-analysis is a valuable and relevant analysis of first-order meta-analysis that provides higher statistical power and efficient evidence for policymakers. While we acknowledge that methodological guidance for meta-meta-analysis is limited, we argue that there is sufficient evidence to apply this method in this context. We consulted methodological research papers^4–6^ and previously published systematic reviews with meta-meta-analysis in Q1 journals^7–9^ to inform our meta-meta-analytic approach.

## **Supplementary text S4: Corrected covered area: formula and interpretation**

Source: Pieper et al. Systematic review finds overlapping reviews were not mentioned in every other overview. *J clinical epidemiology.* 2014;67:368-375. Doi: 10.1016/j.jclinepi.2013.11.007.

$$CCA=\frac{N-r}{rc-r}$$

1: Corrected covered area formula: ‘where *N* is the number of included publications (including double counting) in evidence synthesis (this is the sum of the ticked boxes in the citation matrix); where *r* is the number of rows (number of index publications) and *c* is the number of columns (number of reviews)’ (p.370)

| **CCA** | **Overlap** |
| --- | --- |
| 0-5 | Slight |
| 6-10 | Moderate |
| 11-15 | High |
| >15 | Very High |

2: Interpretation CCA

## **Supplementary text S5: Applying GRADE to Umbrella Reviews**

The Cochrane Handbook for Systematic Reviews and Interventions recommends that overviews of reviews/umbrella reviews assess and report certainty of evidence for each outcome using the GRADE tool^3^. Authors are recommended to extract and report GRADE assessments from each of the included systematic reviews (see eTable 10); however, not all systematic reviews include appropriate GRADE or other certainty of evidence assessments^3^. In this case, authors should complete their own GRADE assessments at review level, so based on the information provided in the included systematic reviews^3,10^.

Based on this guidance, we reported the GRADE assessments, which were only available for 5 out of 19 reviews (eTable 9) and also conducted our own GRADE assessment following available guidance^11–13^. To date, there is no specific checklist or step-by-step guidance to conduct a GRADE assessment in an Umbrella Review context. Even in the Cochrane handbook, authors are recommended to consult Meader at al.’s^13^ guidance for assessing certainty of evidence in systematic reviews. Therefore, we followed the framework for assessing GRADE at primary study level^11–13^ and applied it to the systematic review context. We systematically assessed each of the five GRADE domains (risk of bias, inconsistency, indirectness, imprecision, publication bias)^11^ for the outcome ‘perinatal depressive symptoms’. In line with the available but limited guidance, we assessed each domain based on the information available in each systematic review. For risk of bias, we also took into consideration our risk of bias assessment at overview level.

Applying GRADE in an umbrella review context may pose some limitations. One, there is limited guidance for applying this framework at review level, meaning we had to rely on guidance for applying GRADE to primary studies. While this was possible, concrete guidance for GRADEing Umbrella Reviews is needed to respond to particularities of this systematic review type. Two, staying at review level in our assessment meant that required information was not always available to assess each domain. This may have limited the breadth of our assessment for certain domains. Third, it was unclear to what extent risk of bias or other assessments performed at overview level should be included in the GRADE assessment. After some discussion, we decided to include the AMSTAR-2 rating in our assessment, but further methodological guidance is also needed in this context.

## **Supplementary text S6: Narrative review of physical activity intensity and perinatal depressive symptoms**

Prior to adjusting for overlap, seven reviews assessed the effects of light intensity PA on perinatal depressive symptoms^14–20^. All of the reviews included yoga as a light intensity PA type^14–20^. Five assessed light intensity PA during the prenatal period^14–16,18,20^: one during the postpartum period^17^, and one over the entire perinatal^19^. All but one effect review^19^ found significant effects of light intensity PA on perinatal depressive symptoms, ranging from moderate (SMD: -0∙45 ^20^) to strong (SMD: -1∙10^14^).

Three reviews included moderate intensity PA^17,19,21^, consisting of walking^17,19^ and general aerobic exercise^21^. Wang^17^ identified significant, strong effects for moderate-intensity PA during the postpartum period on postpartum depressive symptoms (SMD: -0∙91). Morres^21^ found significant, moderate small effects (SMD: -0∙43). For moderate-intensity PA during the prenatal period on postpartum depressive symptoms. Liu^19^ did not find significant effects.

Only one review included vigorous-intensity PA^18^, but did not find significant effects on prenatal depressive symptoms.

Eleven reviews^17,19,20,22–29^ did not stratify by PA type, so these were classified as mixed intensity. Three reviews^18,20,23^ focused on outcomes in the prenatal period, five on the postpartum period^17,22,24,25,27^ and three^19,26,28^ on the perinatal period. Most reviews reported significant effects of mixed-intensity PA on perinatal depressive symptoms (SMD: -0∙23 to -0∙74^17,19,20,22,23,26,27,29^); Hicks et al.^24^ reported an MD of 7∙59, but the interpretation of this value remained unclear in the paper. Xu et al.^28^ reported a significant pooled MD of -1.09. McCurdy et al.^25^ did not find significant effects of mixed-PA intensity on perinatal depressive symptoms

## **Supplementary text S7: Narrative review of physical activity type and perinatal depressive symptoms**

The reviews included following PA types: yoga^14–20,23^, walking^17,19^, aerobic exercise^23,28^, and PA with co-intervention^23,27^. The rest did not stratify by PA type and were classified as mixed PA types^17–27^. Effect estimates were varied for each PA type. For yoga, seven reviews reported significant effects on perinatal depressive symptoms (SMD: -0∙23 to -1∙108^14–18,20,23^). Only one review did not report significant effects^19^. Significant, strong effect estimates were reported for walking (SMD: -1∙09^19^; SMD: -0∙91^17^). Effects for aerobic exercise were inconsistent: Xu et al.^28^ did not find significant effects while Davenport et al.^23^ reported significant, weak effects. Similar inconsistencies were reported for PA with co-intervention: Davenport et al.^23^ did not identify significant effects while Pritchett et al.^27^ found significant, weak effects on perinatal depressive symptoms. Reviews predominantly identified a significant association between mixed PA types and perinatal depressive symptoms (SMD: -0∙21 to -0∙77^17–23,25,26^). Pritchett et al.^27^ did not report a significant association. Hicks reported an MD, but the interpretation of this was unclear.

## **Supplementary text S8: Further results on population, supervision, and duration**

Meta-meta-analyses were not feasible for population, supervision, or duration due to variability in reporting and outcomes. Findings on population effects were inconsistent: some reviews reported stronger effects in postpartum or depressed populations^15,25,28^, while others found no significant subgroup differences^27–29^. Timing also showed mixed results, with some reviews identifying effects in both prenatal and postpartum periods, while others found effects only in one phase. One review^21^ suggested potentially greater effects for previously sedentary women.

Regarding supervision, most reviews found no significant difference between supervised and unsupervised interventions. However, one review^19^ reported the highest effects for mixed-mode interventions combining individual and group formats.

Findings on duration were also mixed. While some reviews^18,22^ reported no clear differences, others^19,30^ indicated higher effects for interventions lasting 6-9 weeks or ≥12 weeks, particularly outside of postpartum depression-only populations.

# **Supplementary figures**

## **Supplementary figure S1: Sensitivity analysis: Physical activity intensity and perinatal depressive symptoms**

## **Supplementary figure S2: Sensitivity analysis: Physical activity type and perinatal depressive symptoms**

## **Supplementary figure S3: Meta-meta-analysis: PA and prenatal anxiety symptoms**

## **Supplementary figure S4: Egger’s test: Review pool**

## **Supplementary figure S5: Funnel plot: physical activity and perinatal depressive symptoms**

## **Supplementary figure S6: Funnel plot: physical activity intensity and perinatal depressive symptoms**

## **Supplementary figure S7: Funnel plot: physical activity type and perinatal depressive symptoms**

# **Supplementary figure S8: Funnel plot: physical activity and prenatal anxiety symptoms**

# **Supplementary tables**

## **Supplementary table S1: PICO Table**

| **Population** | Women in the perinatal period (i.e., from the beginning of pregnancy until one year after birth) |
| --- | --- |
| **Intervention** | Any type of physical activity intervention |
| **Comparison** | Any non-PA comparison group (e.g., standard care, waitlist, active control (not PA), no care/no comparison |
| **Outcome** | Perinatal mental health of mothers (i.e., the most common mental health problems in this period: anxiety, depression) |

## **Supplementary table S2: Search strategy**

Recent reviews^31,32^ as well as the Information Specialists' Sub-Group (ISSG) filter recourse^33^ and predefined search strategies for the perinatal period^34,35^ were consulted to develop our search strategy. The search strategy was peer-reviewed using the evidence-based Press Review of Electronic Search Strategies (PRESS) checklist^36^.

**MEDLINE (via PubMed)**

| Population | (“pregnancy”[Mesh] OR pregnan* [tiab] OR perinatal* [tiab] OR peri-natal* [tiab] OR “peri natal*” [tiab] OR “ante natal*” [tiab] OR antenatal* [tiab] OR ante-natal [tiab] OR “pre natal*” [tiab] OR prenatal* [tiab] OR pre-natal* [tiab] OR prepartum [tiab] OR “pre partum” [tiab] OR pre-partum [tiab] OR antepartum [tiab] OR ante-partum [tiab] OR “ante partum” [tiab] OR puerper* [tiab] OR postnatal* [tiab] OR post-natal [tiab] OR postpartum [tiab] OR post-partum [tiab] OR “post partum” [tiab] OR “post natal*” [tiab] OR peripartum [tiab] OR “peri partum” [tiab] OR peri-partum [tiab] OR (expectant* adj2 mother* [tiab]) OR “mother-to-be” [tiab] OR “mothers-to-be” [tiab] OR maternit* [tiab] OR maternal [tiab] OR “before delivery” [tiab] OR postpartal [tiab] OR post-partal [tiab] OR “post partal” [tiab] OR “pregnant women” OR “postpartum period”[Mesh]) |
| --- | --- |
| Exposure | ("Exercise"[Mesh] OR "Sports"[Mesh] OR "Dancing"[Mesh] OR "Motor Activity"[Mesh:NoExp] OR "Physical Exertion"[Mesh] OR "Exercise Movement Techniques"[Mesh] OR ((physical[tiab]) AND (activ*[tiab] OR lifestyle[tiab] OR life-style[tiab] OR "life style"[tiab])) OR ((exercise[tiab]) AND (train*[tiab] OR physical*[tiab] OR active*[tiab])) OR exercise*[tiab] OR sport*[tiab] OR "physical exertion*" OR "motor activ*"[tiab] OR "train*"[tiab] OR "physical effort*"[tiab] OR "physical endurance"[tiab] OR "physical condition*"[tiab] OR gymnastic*[tiab] OR run[tiab] OR runn*[tiab] OR swim*[tiab] OR walk*[tiab] OR jogg*[tiab] OR athletic*[tiab] OR cycl*[tiab] OR bicycl*[tiab] OR aerobic*[tiab] OR move[tiab] OR yoga[tiab] OR strength*[tiab] OR resistance*[tiab] OR “weight lifting”[tiab] OR pilates[tiab] OR “exercise therapy”[tiab] OR step*[tiab] OR (“tai ji”[tiab] OR “tai chi”[tiab] OR tai-ji[tiab] OR tai-chi[tiab])) |
| Comparison | Mothers in the perinatal period with a non-PA comparison/standard care/waitlist/nothing (will be screened manually) |
| Outcome | (“Mental health”[Mesh] OR “Mental disorders”[Mesh] OR “depression”[Mesh] OR “depressive disorder”[Mesh] OR “depression, postpartum”[Mesh] OR “perinatal depression” [tiab] OR depressi* [tiab] OR “anxiety”[Mesh] OR “Anxiety disorders”[Mesh] OR anxi* [tiab]) |
| Publication type | Following search string adapted from <https://www.ncbi.nlm.nih.gov/pmc/articles/PMC8608217/>:  ((systematic* [tiab] AND review [tiab]) OR Systematic overview* [tiab] OR Cochrane review* [tiab] OR systemic review* [tiab] OR Systematic literature search [tiab] OR Systematic literature research [tiab] OR ((search* [tiab] OR medline [tiab] OR pubmed [tiab] OR embase [tiab] OR Cochrane [tiab] OR scopus [tiab] or web of science [tiab] OR sources of information [tiab] OR data sources [tiab] OR following databases [tiab]) AND (study selection [tiab] OR selection criteria [tiab] OR eligibility criteria [tiab] OR inclusion criteria [tiab] OR exclusion criteria [tiab])) OR systematic review [pt])  AND medline[sb] |
| Language | (english[la] OR german[la]) |
| Time frame | (2014:2024[dp]) |

**MEDLINE/EMBASE (via Embase)**

| Population | pregnan*:ab,ti OR perinatal*:ab,ti OR peri-natal*: ab,ti OR ‘peri natal*’:ab,ti OR ‘ante natal*’:ab,ti OR antenatal*:ab,ti OR ante-natal:ab,ti OR ‘pre natal*’:ab,ti OR prenatal*:ab,ti OR pre-natal*:ab,ti OR prepartum:ab,ti OR ‘pre partum’:ab,ti OR pre-partum:ab,ti OR antepartum:ab,ti OR ante-partum:ab,ti OR ‘ante partum’:ab,ti OR puerper*:ab,ti OR postnatal*:ab,ti OR post-natal:ab,ti OR postpartum:ab,ti OR post-partum:ab,ti OR ‘post partum’:ab,ti OR ‘post natal*’:ab,ti OR peripartum:ab,ti OR ‘peri partum’:ab,ti OR peri-partum:ab,ti OR (expectant* adj2 mother*:ab,ti) OR ‘mother-to-be’:ab,ti OR ‘mothers-to-be’:ab,ti OR maternit*:ab,ti OR maternal:ab,ti OR ‘before delivery’:ab,ti OR postpartal:ab,ti OR post-partal:ab,ti OR ‘post partal’:ab,ti |
| --- | --- |
| Intervention | 'exercise'/exp OR 'physical activity'/exp OR 'sport'/exp OR 'dancing'/exp OR 'motor activity'/exp OR ((physical:ab,ti) AND (activ*:ab,ti OR lifestyle:ab,ti OR 'life style':ab,ti)) OR ((exercise:ab,ti) AND (train*:ab,ti OR physical*:ab,ti OR active*:ab,ti)) exercise*:ab,ti OR sport*:ab,ti OR 'physical exertion*' OR 'motor activ*':ab,ti OR 'train*':ab,ti OR 'physical effort*':ab,ti OR 'physical endurance':ab,ti OR 'physical condition*':ab,ti OR gymnastic*:ab,ti OR run:ab,ti OR runn*:ab,ti OR swim*:ab,ti OR walk*:ab,ti OR jogg*:ab,ti OR athletic*:ab,ti OR cycl*:ab,ti OR aerobic*:ab,ti OR move:ab,ti OR bicycl*:ab,ti OR yoga:ab,ti OR strength*:ab,ti OR resistance*:ab,ti OR “weight lifting”:ab,ti OR pilates:ab,ti OR ‘exercise therapy’:ab,ti OR step*:ab,ti OR (‘tai ji’:ab,ti OR ‘tai chi’:ab,ti OR tai-ji:ab,ti OR tai-chi:ab,ti) |
| Comparison | Mothers in the perinatal period with a non-PA comparison/standard care/waitlist/nothing (will be screened manually) |
| Outcome | ‘Mental health’/exp OR ‘Mental disorders’/exp OR ‘depression’/exp OR ‘depressive disorder’/exp OR ‘postpartum depression’:ab,ti OR ‘perinatal depression’:ab,ti OR depressi*:ab,ti OR anxiety:ab,ti OR ‘anxiety disorders’:ab,ti OR anxi*:ab,ti |
| Publication type | ((systematic*:ti,ab AND review:ti,ab) OR Systematic overview*:ti,ab OR Cochrane review*:ti,ab OR systemic review*:ti,ab OR Systematic literature search:ti,ab OR Systematic literature research:ti,ab OR ((search*:ti,ab OR medline:ti,ab OR pubmed:ti,ab OR embase:ti,ab OR Cochrane:ti,ab OR scopus:ti,ab or web of science:ti,ab OR sources of information:ti,ab OR data sources:ti,ab OR following databases:ti,ab) AND (study selection:ti,ab OR selection criteria:ti,ab OR eligibility criteria:ti,ab OR inclusion criteria:ti,ab OR exclusion criteria:ti,ab)) OR 'systematic review'/exp) |
| Language | (English/la OR german/la) |
| Time frame | [2014-2024]/py |

**Web of Science (via Clarivate)**

| Population | Ts=(pregnan* OR perinatal* OR peri-natal* OR “peri natal*” OR “ante natal*” OR antenatal* OR ante-natal OR “pre natal*” OR prenatal* OR pre-natal* OR prepartum OR “pre partum” OR pre-partum OR antepartum OR ante-partum OR “ante partum” OR puerper* OR postnatal* OR post-natal OR postpartum OR post-partum OR “post partum” OR “post natal*” OR peripartum OR “peri partum” OR peri-partum OR (expectant* NEAR/5 mother*) OR “mother-to-be” OR “mothers-to-be” OR maternit* OR maternal OR “before delivery” OR postpartal OR post-partal OR “post partal”)  Alternative:  Ts=(pregnan* OR perinatal* OR peri-natal* OR antenatal* OR ante-natal OR prenatal* OR pre-natal* OR postnatal* OR post-natal OR postpartum OR post-partum OR (expectant* NEAR/5 mother*) OR maternit* OR maternal) |
| --- | --- |
| Intervention | Ts=(((physical) AND (activ* OR lifestyle OR life-style OR "life style")) ((exercise) AND (train* OR physical* OR active*)) OR exercise* OR sport* OR "physical exertion*" OR "motor activ*" OR "train*" OR "physical effort*" OR "physical endurance" OR "physical condition*" OR gymnastic* OR run OR runn* OR swim* OR walk* OR jogg* OR athletic* OR cycl* OR aerobic* OR move OR bicycl* OR yoga OR strength* OR resistance* OR “weight lifting” OR pilates OR “exercise therapy” OR step* OR (“tai ji” OR “tai chi” OR tai-ji OR tai-chi)) |
| Comparison | Mothers in the perinatal period with a non-PA comparison/standard care/waitlist/nothing (will be screened manually) |
| Outcome | Ts=(“Mental health” OR “Mental disorders” OR depressi* OR “depressive disorder” OR “postpartum depression” OR “perinatal depression” OR “Anxiety disorders” OR anxi*) |
| Publication type | Ts=((systematic*AND review) OR Systematic overview* OR Cochrane review* OR systemic review* OR Systematic literature search OR Systematic literature research) |
| Language | English or German – this will be screened manually |
| Time frame | PY=(2014-2024) |

**APA PsycInfo (via EBSCOhost)**

| Population | (“perinatal period” OR “postnatal period” OR postnatal OR perinatal OR postpartum OR “postpartum period” OR maternity OR “antepartum period” OR “expectant mothers” OR maternal OR pregnan* OR perinatal* OR peri-natal* OR “peri natal*” OR “ante natal*” OR antenatal* OR ante-natal OR “pre natal*” OR prenatal* OR pre-natal* OR prepartum OR “pre partum” OR pre-partum OR antepartum OR ante-partum OR “ante partum” OR puerper* OR postnatal* OR post-natal OR postpartum OR post-partum OR “post partum” OR “post natal*” OR peripartum OR “peri partum” OR peri-partum OR “mother-to-be” OR “mothers-to-be” OR maternit* OR maternal OR “before delivery” OR postpartal OR post-partal OR “post partal” ) |
| --- | --- |
| Intervention | (exercise OR sports OR “motor activity OR “physical activity” OR training OR OR sport* OR "physical exertion" OR "motor activ*" OR "physical effort*" OR "physical endurance" OR "physical condition*" OR gymnastic* OR run OR runn* OR swim* OR walk* OR jogg* OR athletic* OR cycl* OR bicycl* OR aerobic* OR move OR yoga OR strength* OR resistance* OR “weight lifting” OR pilates OR “exercise therapy” OR step* OR “tai ji” OR “tai chi” OR tai-ji OR tai-chi) |
| Comparison | Mothers in the perinatal period with a non-PA comparison/standard care/waitlist/nothing (will be screened manually) |
| Outcome | (“postnatal depression” OR “postpartum blues” OR “postpartum emotional disturbance” OR “puerperal depression” OR “maternity blues” OR “mental health” OR “mental disorders” OR depressi* OR “depressive disorder” OR “major depression” OR anxi* OR “anxiety disorder” OR “perinatal depression”) |
| Publication type | (PT SR OR MA)Po |
| Language | (LA English OR LA German) |
| Time Frame | YR 2014-2024 |

**Cochrane (via cochranelibrary.com)**

| Population | #1 MeSH descriptor: [pregnancy] this term only  #2 MeSH descriptor: [pregnant women] explode all trees  #3 MeSh descriptor: [postpartum period] explode all trees  #4 (pregnan* OR perinatal* OR peri-natal* OR (peri NEXT natal*) OR (ante NEXT natal*) OR antenatal* OR ante-natal OR (pre NEXT natal*) OR prenatal* OR pre-natal* OR prepartum OR “pre partum” OR pre-partum OR antepartum OR ante-partum OR “ante NEXT partum” OR puerper* OR postnatal* OR post-natal OR postpartum OR post-partum OR “post NEXT partum” OR (post NEXT natal*) OR peripartum OR “peri NEXT partum” OR peri-partum OR (expectant* NEXT mother*) OR “mother-to-be” OR “mothers-to-be” OR maternit* OR maternal OR “before NEXT delivery” OR postpartal OR post-partal OR “post NEXT partal”):ti,kw,ab  #5 #1 OR #2 OR #3 OR #4 |
| --- | --- |
| Intervention | #6 MeSH descriptor: [Exercise] explode all trees  #7 MeSH descriptor: [Sports] explode all trees  #8 MeSH descriptor: [Dancing] explode all trees  #9 MeSH descriptor: [Motor Activity] explode all trees  #10 MeSH descriptor: [Physical Exertion] explode all trees  #11 MeSH descriptor: [Exercise Movement Techniques] explode all trees  #12 (((physical) AND (activ* OR lifestyle OR life-style OR (life NEXT style))) OR ((exercise) AND (train* OR physical* OR active*)) OR exercise* OR sport* OR (physical NEXT exertion*) OR (motor NEXT activ*) OR train* OR (physical NEXT effort*) OR (physical NEXT endurance) OR (physical NEXT condition*) OR gymnastic* OR run OR runn* OR swim* OR walk* OR jogg* OR athletic* OR cycl* OR aerobic* OR move OR bicycl* OR yoga OR strength* OR resistance* OR (weight NEXT lifting) OR pilates OR (exercise NEXT therapy) OR step* OR ((tai NEXT ji) OR (tai NEXT chi) OR tai-ji OR tai-chi)):ti,ab,kw (Word variations have been searched)  #13 #7 OR #8 OR #9 OR #10 OR #11 OR #12  #14 #5 AND #13 |
| Comparison | Mothers in the perinatal period with a non-PA comparison/standard care/waitlist/nothing (will be screened manually) |
| Outcome | #15 MeSH descriptor: [mental health] explode all trees  #16 MeSH descriptor: [mental disorders] explode all trees  #17 MeSH descriptor: [depression] explode all trees  #18 MeSH descriptor: [depressive disorder] explode all trees  #19 MeSH descriptor: [depression, postpartum] explode all trees  #20 MeSH descriptor: [anxiety] explode all trees  #21 MeSH descriptor: [anxiety disorders] explode all trees  #22 (depressi* OR anxi*):ti,kw,ab  #23 #17 OR #18 OR #19 OR #20 OR #21 OR #22  #24 #14 AND #23 |
| Publication type | #25 (systematic review):pt  #26 #24 AND #25 |
| Language | English or German (will be screened manually) |
| Time Frame | Date range will be searched manually |

**Epistemonikos (via epistemonikos.com)**

| Population | (title:(pregnan* OR perinatal* OR peri-natal* OR “peri natal*” OR “ante natal*” OR antenatal* OR ante-natal OR “pre natal*” OR prenatal* OR pre-natal* OR prepartum OR “pre partum” OR pre-partum OR antepartum OR ante-partum OR “ante partum” OR puerper* OR postnatal* OR post-natal OR postpartum OR post-partum OR “post partum” OR “post natal*” OR peripartum OR “peri partum” OR peri-partum OR (expectant* NEAR/5 mother*) OR “mother-to-be” OR “mothers-to-be” OR maternit* OR maternal OR “before delivery” OR postpartal OR post-partal OR “post partal”)  OR abstract:(pregnan* OR perinatal* OR peri-natal* OR “peri natal*” OR “ante natal*” OR antenatal* OR ante-natal OR “pre natal*” OR prenatal* OR pre-natal* OR prepartum OR “pre partum” OR pre-partum OR antepartum OR ante-partum OR “ante partum” OR puerper* OR postnatal* OR post-natal OR postpartum OR post-partum OR “post partum” OR “post natal*” OR peripartum OR “peri partum” OR peri-partum OR (expectant* NEAR/5 mother*) OR “mother-to-be” OR “mothers-to-be” OR maternit* OR maternal OR “before delivery” OR postpartal OR post-partal OR “post partal”)) |
| --- | --- |
| Intervention: Practice (i.e., intervention, prevention, healthcare, guidelines) | (title:(((physical) AND (activ* OR lifestyle OR life-style OR "life style")) OR ((exercise) AND (train* OR physical* OR active*)) OR exercise* OR sport* OR "physical exertion*" OR "motor activ*" OR "train*" OR "physical effort*" OR "physical endurance" OR "physical condition*" OR gymnastic* OR run OR runn* OR swim* OR walk* OR jogg* OR athletic* OR cycl* OR aerobic* OR move OR bicycl* OR yoga OR strength* OR resistance* OR “weight lifting” OR pilates OR “exercise therapy” OR step* OR (“tai ji” OR “tai chi” OR tai-ji OR tai-chi)))  OR (abstract:(((physical) AND (activ* OR lifestyle OR life-style OR "life style")) OR ((exercise) AND (train* OR physical* OR active*)) OR exercise* OR sport* OR "physical exertion*" OR "motor activ*" OR "train*" OR "physical effort*" OR "physical endurance" OR "physical condition*" OR gymnastic* OR run OR runn* OR swim* OR walk* OR jogg* OR athletic* OR cycl* OR aerobic* OR move OR bicycl* OR yoga OR strength* OR resistance* OR “weight lifting” OR pilates OR “exercise therapy” OR step* OR (“tai ji” OR “tai chi” OR tai-ji OR tai-chi))) |
| Comparison | N/A |
| Outcome | (title:(“Mental health” OR “Mental disorders” OR depressi* OR “depressive disorder” OR “postpartum depression” OR “perinatal depression” OR “Anxiety disorders” OR anxi*)) OR (abstract:(“Mental health” OR “Mental disorders” OR depressi* OR “depressive disorder” OR “postpartum depression” OR “perinatal depression” OR “Anxiety disorders” OR anxi*)) |
| Publication type | (title:((systematic*AND review) OR “Systematic overview*” OR “Cochrane review*” OR “systemic review*” OR “Systematic literature search” OR “Systematic literature research”)) OR (abstract:((systematic*AND review) OR Systematic overview*OR Cochrane review* OR systemic review* OR “Systematic literature search” OR “systematic literature research”))  Search filter for systematic reviews |
| Language | English or German, will be screened manually |
| Time Frame | 2014-2024, time frame will be screened manually |

**DoPHER (via EPPI-centre)**

| Population | (“pregnan*”) OR (“perinatal*”) OR (“peri-natal*”) OR (“peri natal*”) OR (“ante natal*”) OR (“antenatal*”) OR ante-natal OR (“pre natal*”) OR (“prenatal*”) OR (“pre-natal*”) OR prepartum OR “pre partum” OR pre-partum OR antepartum OR ante-partum OR “ante partum” OR (“puerper*”) OR (“postnatal*”) OR post-natal OR postpartum OR post-partum OR “post partum” OR (“post natal*”) OR peripartum OR “peri partum” OR peri-partum OR (“expectant* NEAR/5 mother*”) OR “mother-to-be” OR “mothers-to-be” OR (“maternit*”) OR maternal OR “before delivery” OR postpartal OR post-partal OR “post partal” |
| --- | --- |
| Intervention: Practice (i.e., intervention, prevention, healthcare, guidelines) | ((physical) AND (“activ*” OR lifestyle OR life-style OR "life style")) OR ((exercise) AND (“train*” OR “physical*” OR “active*”)) OR “exercise*” OR “sport*” OR "physical exertion*" OR "motor activ*" OR "train*" OR "physical effort*" OR "physical endurance" OR "physical condition*" OR “gymnastic*” OR run OR “runn*” OR “swim*” OR “walk*” OR “jogg*” OR “athletic*” OR “cycl*” OR “aerobic*” OR move OR “bicycl*” OR yoga OR “strength*” OR “resistance*” OR “weight lifting” OR pilates OR “exercise therapy” OR “step*” OR (“tai ji” OR “tai chi” OR tai-ji OR tai-chi))  Alternative: |
| Comparison | N/A |
| Outcome | (title:(“Mental health” OR “Mental disorders” OR (“depressi*”) OR “depressive disorder” OR “postpartum depression” OR “perinatal depression” OR “Anxiety disorders” OR (“anxi*”))) OR (abstract: (“Mental health” OR “Mental disorders” OR (“depressi*”) OR “depressive disorder” OR “postpartum depression” OR “perinatal depression” OR “Anxiety disorders” OR (“anxi*”))) |
| Publication type | Search filter for systematic reviews (*search string for SRs causes difficulties in ePPI search)* |
| Language | English or German, will be screened manually |
| Time Frame | 2014-2024, time frame will be screened manually |

**CINAHL (via EBSCOhost)**

| Population | (pregnan* OR perinatal* OR peri-natal* OR “peri natal*” OR “ante natal*” OR antenatal* OR ante-natal OR “pre natal*” OR prenatal* OR pre-natal* OR prepartum OR “pre partum” OR pre-partum OR antepartum OR ante-partum OR “ante partum” OR puerper* OR postnatal* OR post-natal OR postpartum OR post-partum OR “post partum” OR “post natal*” OR peripartum OR “peri partum” OR peri-partum OR “mother-to-be” OR “mothers-to-be” OR maternit* OR maternal OR “before delivery” OR postpartal OR post-partal OR “post partal” ) |
| --- | --- |
| Intervention | (((physical) AND (activ* OR lifestyle OR life-style OR "life style")) OR ((exercise) AND (train* OR physical* OR active*)) OR exercise* OR sport* OR "physical exertion*" OR "motor activ*" OR "train*" OR "physical effort*" OR "physical endurance" OR "physical condition*" OR gymnastic* OR run OR runn* OR swim* OR walk* OR jogg* OR athletic* OR cycl* OR bicycl* OR aerobic* OR move OR yoga OR strength* OR resistance* OR "weight lifting" OR pilates OR "exercise therapy" OR step* OR ("tai ji" OR "tai chi" OR tai-ji OR tai-chi)) |
| Comparison | Mothers in the perinatal period with a non-PA comparison/standard care/waitlist/nothing (will be screened manually) |
| Outcome | "mental health" OR "perinatal depression" OR depressi OR anxi |
| Publication type | "systematic review" OR "meta-analysis" |
| Language | (LA English OR LA German) |
| Time Frame | YR 2014-2024 |

**Sports Medicine & Education Index (via ProQuest)**

| Population | (pregnan* OR perinatal* OR peri-natal* OR “peri natal*” OR “ante natal*” OR antenatal* OR ante-natal OR “pre natal*” OR prenatal* OR pre-natal* OR prepartum OR “pre partum” OR pre-partum OR antepartum OR ante-partum OR “ante partum” OR puerper* OR postnatal* OR post-natal OR postpartum OR post-partum OR “post partum” OR “post natal*” OR peripartum OR “peri partum” OR peri-partum OR “mother-to-be” OR “mothers-to-be” OR maternit* OR maternal OR “before delivery” OR postpartal OR post-partal OR “post partal” ) |
| --- | --- |
| Intervention | (((physical) AND (activ* OR lifestyle OR life-style OR "life style")) OR ((exercise) AND (train* OR physical* OR active*)) OR exercise* OR sport* OR "physical exertion*" OR "motor activ*" OR "train*" OR "physical effort*" OR "physical endurance" OR "physical condition*" OR gymnastic* OR run OR runn* OR swim* OR walk* OR jogg* OR athletic* OR cycl* OR bicycl* OR aerobic* OR move OR yoga OR strength* OR resistance* OR "weight lifting" OR pilates OR "exercise therapy" OR step* OR ("tai ji" OR "tai chi" OR tai-ji OR tai-chi)) |
| Comparison | Mothers in the perinatal period with a non-PA comparison/standard care/waitlist/nothing (will be screened manually) |
| Outcome | "mental health" OR "perinatal depression" OR depressi OR anxi |
| Publication type | "systematic review" OR "meta-analysis" |
| Language | English or German, will be screened manually |
| Time Frame | 2014-2024, time frame will be screened manually |

## **Supplementary table S3: Reasons for exclusion of studies from the systematic literature search, after screening for title and abstract**

| **Study** | **Reason for exclusion** |
| --- | --- |
| Christian et al. (2022) | Publication status (dissertation) |
| Corrigan et al. (2019) | Population (included high-risk pregnant women) |
| Deprato et al. (2024) | No response from authors |
| Dong et al. (2026 | Study design (use of different measurement instruments) |
| Evans et al. (2018) | Study design (only non-randomized interventions) |
| Fuenzalida et al. (2025) | Values in synthesis could not be validated |
| Gillinsky et al. (2015) | Study design (does not fulfil meta-analysis criteria) |
| Jiang et al. (2025) | Study design (credible intervals) |
| Lin et al. (2022) | Study design (does not fulfil meta-analysis criteria) |
| Liu et al. (2023) | Comparison (included exercise) |
| Liu et al. (2025) | Intervention (yoga combined with other mind-body-interventions) |
| Mai et al. (2023) | Comparison (included psychological care) |
| Marconcin et al. (2021) | Study design (does not fulfil meta-analysis criteria) |
| Moufang et al. (2024) | Full text could not be retrieved |
| Ningrum et al. (2019) | No risk of bias assessment |
| Poyatos-León et al. (2017) | Study design (did not fulfil criteria for meta-analysis) |
| Punsuwun et al. (2025) | Study design (RCTs mixed with other study designs) |
| Ranjbar et al. (2026) | Study design (RCTs & quasi-experimental) |
| Regan et al. (2015) | Publication type (magazine article) |
| Ruan et al. (2025) | Intervention (yoga combined with psychological interventions) |
| Sánchez-Polán et al. (2021a) | Study design (did not fulfil criteria for meta-analysis) |
| Sánchez-Polán et al. (2021b) | Intervention |
| Shuai et al. (2024) | Study design (does not fulfil meta-analysis criteria) |
| Smith et al. (2025) | Study design (credible intervals) |
| Wang et al. (2023) | Comparison (includes exercise) |
| Xie et al. (2025) | Study design (MA effect estimates cannot be used in our study) |
| Zeng et al. (2025) | Study design (missing studies) |
| Zhao et al. (2024) | Outcome |
| Zimmerman et al. (2023) | Intervention (psychological intervention) |
| Zhu et al. (2025) | Study design (credible intervals) |

## **Supplementary table S4: PRIOR checklist for overviews of reviews (Gates et al., 2022)**

## **Supplementary table S5: Fusar-Poli and Radua’s (2018) ‘Ten simple rules for conducting umbrella reviews’**

Source: Fusar-Poli P, Radua J. Ten simple rules for conducting umbrella reviews. *Evidence Based Mental Health*. 2018;21:. <https://doi.org/10.1136/ebmental-2018-300014>.

| 1 | Ensure that the umbrella review is really needed |
| --- | --- |
| 2 | Prespecify the protocol |
| 3 | Clearly define the variables of interest |
| 4 | Estimate a common effect size |
| 5 | Report the heterogeneity and potential biases |
| 6 | Perform a stratification of evidence |
| 7 | Conduct (study-level) sensitivity analyses |
| 8 | Report transparent results |
| 9 | Use appropriate software |
| 10 | Acknowledge its limitations |

## **Supplementary table S6: Deviations from the protocol**

| **Section of protocol** | **Original plan** | **Deviation** | **Reason for change** | **Timing (before or after data extraction)** |
| --- | --- | --- | --- | --- |
| Eligibility criteria | Outcomes: depression, anxiety, stress, trauma, fatigue | Removed stress, trauma, fatigue | Lack of RCTs on these outcomes | Before |
| Strategy for data synthesis | Meta-meta-analysis for perinatal depression and anxiety | Only for perinatal depressive symptoms | Not enough reviews available and overlap too high to pool | After |
| Strategy for data synthesis | No mention of overlap calculations | Included overlap calculations using CCA and grouped reviews according to topic to analyze | Reduced potential bias in study results | After |
| Strategy for data synthesis | Report findings using evidence heat maps and forest plots | Only forest plots | Too few effect sizes to create an evidence heat map | After |

## **Supplementary table S7: Detailed AMSTAR-2 risk of bias assessment**

## **Supplementary table S8: Overview of studies for meta-meta-analysis and overlap adjustment**

## **Supplementary table S9: Physical activity intensity MET value cut-offs**

Source: Stephen D. Herrmann, Erik A. Willis, Barbara E. Ainsworth, Tiago V. Barreira, Mary Hastert, Chelsea L. Kracht, John M. Schuna, Zhenghua Cai, Minghui Quan, Catrine Tudor-Locke, Melicia C. Whitt-Glover, David R. Jacobs. Adult Compendium of Physical Activities: A third update of the energy costs of human activities. *Journal of Sport and Health Science*. 2024.

| **MET Value** | **Intensity** |
| --- | --- |
| 1.0-1.5 METs | Sedentary |
| 1.6-2.9 METs | Light |
| 3.0-5.9 METs | Moderate |
| ≥6 METs | Vigorous |

Physical activity MET Values based on the 2024 Compendium of Adult Physical Activities

| **Physical Activity Type** | **MET Value** |
| --- | --- |
| Yoga, general | 2.3 |
| Aerobic, general | 7.3 |
| Walking, for pleasure | 3.5 |

## **Supplementary table S10: Reporting of GRADE assessments in the included systematic reviews**

| **Author** | **Outcome** | **Domain 1: Risk of Bias** | **Domain 2:**  **Inconsistency** | **Domain 3:**  **Indirectness** | **Domain 4:**  **Imprecision** | **Domain 5:**  **Publication bias/Other considerations** | **Certainty of evidence** |
| --- | --- | --- | --- | --- | --- | --- | --- |
| Carter et al. | Prenatal depressive symptoms | Serious | Not serious | Not serious | Serious | None | LOW |
| Daley et al. | Prenatal depression | N/A | N/A | N/A | N/A | N/A | N/A |
| Davenport et al. | Prenatal depressive symptoms | serious | serious | serious | Not serious | None | VERY LOW |
| Davenport et al. | Prenatal depression | serious | serious | serious | Not serious | None | VERY LOW |
| Davenport et al. | Postnatal depressive symptoms | serious | Not serious | serious | Not serious | None | LOW |
| Davenport et al. | Postnatal depression | serious | Not serious | serious | serious | None | VERY LOW |
| Gong et al. | Prenatal depression | N/A | N/A | N/A | N/A | N/A | N/A |
| He et al. | Perinatal depression | N/A | N/A | N/A | N/A | N/A | N/A |
| Hicks et al. | Postpartum depression and anxiety | N/A | N/A | N/A | N/A | N/A | N/A |
| Liu et al. | Perinatal depressive symptoms | N/A | N/A | N/A | N/A | N/A | LOW -MODERATE |
| McCurdy et al. | Postpartum depressive symptoms | N/A | N/A | N/A | N/A | N/A | N/A |
| Morres et al. | Perinatal depressive symptoms | N/A | N/A | N/A | N/A | N/A | N/A |
| Ji et al. | Perinatal depression | Downgraded one level | No downgrading | No downgrading | No downgrading | No downgrading | MODERATE |
| Nakamura et al. | Postpartum depression | N/A | N/A | N/A | N/A | N/A | N/A |
| Ng et al. | Prenatal depression | N/A | N/A | N/A | N/A | N/A | N/A |
| Pentland et al. | Postpartum depressive symptoms | N/A | N/A | N/A | N/A | N/A | N/A |
| Pritchett et al. | Postpartum depressive symptoms | N/A | N/A | N/A | N/A | N/A | N/A |
| Wang et al. | Postpartum depression | serious | Not serious – serious | Not serious | Not serious | None | LOW - MODERATE |
| Xu et al. | Postpartum depression | N/A | N/A | N/A | N/A | N/A | N/A |
| Yuan et al. | Postpartum depression | N/A | N/A | N/A | N/A | N/A | N/A |
| Zhang et al. | Prenatal depression | N/A | N/A | N/A | N/A | N/A | N/A |
| Zhu et al. | Prenatal depression | N/A | N/A | N/A | N/A | N/A | LOW |
| Miao et al. | Prenatal depression | N/A | N/A | N/A | N/A | N/A | N/A |

## **Supplementary table S11: Perinatal mental health outcome measurement tools**

| **Tool** | **Info** | **Scoring** | **Total Reviews** |
| --- | --- | --- | --- |
| Beck Depression Inventory (BDI; revised: BDI-2) | 21-item self-report aimed at measuring the severity of depression  (BDI-II developed in response to changed diagnostic criteria for depression) | BDI-II:   - 0-13: minimal - 14-19: mild - 20-28: moderate - 29-63: severe | 5 |
| Center for Epidemiological Studies - Depression (CES-D) | 20-item self-report depression scale aimed at screening for symptoms of depression in the general population (especially in older adults) | - ≥16: indicative of depressive symptoms | 15 |
| Depression Anxiety Stress Scale (DASS, DASS-21) | 42-item or 21-item self-report scale aimed at measuring emotional states of depression, anxiety and stress | Depression   - 0-9: normal - 10-13: mild - 14-20 moderate - 21-27: severe   Anxiety   - 0-7: anxiety - 8-9: mild - 10-14: moderate - 15-19: severe | 3 |
| Derogatis Affects Balance Scale (DABS) | 40-item self-report mood inventory aimed at assessing positive and negative affect | - No defined cut-offs | 1 |
| Edinburgh Postnatal Depression Scale (EPDS) | 10-item self-report screening tool (questionnaire) aimed at identifying women at risk of postnatal depression | - Maximum: 30 - Possible depression: 10 or greater - >13: likely to be suffering from depression of varying severity | 20 |
| Hamilton Rating Scale for Depression (HRSD, HDRS, HDS) / Hamilton Scale (HAMD, HAM-D) | 17-item clinician-rated/interviewer-rated scale aimed at measuring severity and frequency of depression symptoms in adults with diagnosed depression | - 0-7: within the normal range or clinical remission - ≥20: at least moderate severity (required for entry into clinical trial) | 8 |
| Hospital Anxiety and Depression Scale (HADS) | 14-item self-report questionnaire aimed at screening adults for depression and anxiety | - 0-7: normal range - 8-10: suggestive of presence of depression and anxiety - ≥11: probable presence of depression and anxiety | 6 |
| Hamilton Anxiety Rating Scale (HAM-A) | 14-item clinician-rated/interviewer-rated scale aimed at measuring severity of symptoms of anxiety | - <17: mild severity - 18-24: mild to moderate - 25-30: moderate to severe | 2 |
| Inventory of Depression and Anxiety Symptoms (IDAS) | 64-item self-report instrument aimed at assessing symptoms of depression and anxiety disorders, using 10 specific symptom domains | - No specifically defined cut-offs - Higher scores indicate higher levels of symptomology | 1 |
| Positive and Negative Affect Schedule (PANAS) | 20-item self-report questionnaire to assess positive and negative affect | - No specifically defined cut-offs - Higher score indicates higher positive or negative affect | 1 |
| Patient health questionnaire (PHQ-9) | 9-item questionnaire aimed at monitoring severity of depression and response to treatment in adults | - 0-4: normal - 5-9: mild - 10-14: moderate - 15-19: moderately severe - 20-27: severe | 3 |
| Perceived Stress Scale (PSS) | 10-item self-report questionnaire aimed at assessing individual stress levels | - 0-13: low stress - 14-26: moderate stress - 27-40: high stress | 1 |
| Psychological general well-being index (PGWBI) | 22-item self-report questionnaire aimed at assessing perceived well-being and psychological distress | - 0-60: severe distress - 61-72: moderate distress - 73-110: no distress | 1 |
| Pregnancy-related anxiety questionnaire (PRAQ) | 34-item questionnaire aimed at assessing anxiety specifically related to pregnancy | - No specifically defined cut-offs - Higher score indicates greater pregnancy-related anxiety | 1 |
| Profile of Mood States (POMS) | 65-item self-report questionnaire measuring six dimensions of mood aimed at assessing total mood disturbance | - No specifically defined cut-offs - Higher scores indicate higher levels of mood disturbance | 4 |
| Quick Inventory of Depressive Symptomology (QIDS) | 16-item self-report questionnaire aimed at assessing severity of depressive symptoms in adults | - 0-5: normal - 6-10: mild - 11-15: moderate - 16-20: severe - 21-27: very severe | 1 |
| State-Trait Anxiety Inventory (STAI) | 40-item self-report questionnaire aimed at assessing trait and state anxiety respectively | - No specifically defined cut-offs - Higher scores indicate higher levels of anxiety | 4 |
| Zung Self-rating Depression Scale (SDS) | 20-item self-report scale aimed at assessing the presence and severity of depression symptoms in adults | - 25-49: normal - 50-59: mild - 60-69: moderate - ≥70: severely depressed | 3 |
| Zung Self-rating Anxiety Scale (SAS) | 20-item self-report scale aimed at assessing the presence and severity of anxiety in adults | - 20-24: normal - 45-59: mild to moderate - 60-74: severe - ≥75: extreme | 2 |
| Symptom Checklist-90 (SCL-90) | 90-item questionnaire aimed at assessing psychological problems | - No specifically defined cut-offs - Higher scores indicate higher symptom severity or psychological distress | 1 |
| Clinical diagnosis/structured diagnosis interview (e.g., SCID) | N/A | N/A | 4 |

## **Supplementary table S12: Reasons for exclusion of meta-analyses**

| **Study** | **Reason for exclusion** |
| --- | --- |
| He et al. (2023) | Overuse of subgroups: Excessive use of subgroups increases the likelihood of finding an effect; however, this will likely be by chance rather than a true effect, which led us to remove these meta-analyses from our meta-meta-analysis. |
| Ji et al. (2024) | Values in the synthesis could not be verified: We checked the primary studies and found that they could either not be located for further information or that the extracted values in the forest plot did not match those in the primary studies. |
| Pentland et al. (2022) | Values in the synthesis could not be verified: Effect sizes in the forest plot did not match those in the primary studies. |
| Yuan et al. (2022) | Unclear effect sizes: One primary study (Ana et al) was classified as an RCT, although it is a cohort study, which may have affected the overall effect calculated for RCTs. |

## **Supplementary table S13: Summary of main findings for perinatal depressive symptoms**

|  | **N** | **n** | **SMD(95% CI)** | | **Effect size**  **P value** | | **Heterogeneity I^2^** | |
| --- | --- | --- | --- | --- | --- | --- | --- | --- |
| Perinatal depressive symptoms | | | | | | | | |
| **Overall PA** | 5 | 9,081 | -0.56 (-0.93; -0.19) | | P = 0.006 | | 72% | |
| **Light-intensity PA** | 4 | 1,172 | -0.52 (-0.81; -0.23) | | P = 0.32 | | 14.5% | |
| **Moderate-intensity PA** | 1 | 296 | -0.43 (-0.64; -0.22) | | NA | | NA | |
| **Vigorous-intensity PA** | 1 | 356 | -0.23 (-0.62; 0.16) | | NA | | NA | |
| **Mixed-intensity PA** | 5 | 6,806 | -0.52 (-0.8; -0.24) | | P = 0.02 | | 65.4% | |
| **Yoga** | 3 | 967 | -0.55 (-0.95; -0.15) | | P = 0.03 | | 70.6% | |
| **Walking** | 1 | 155 | -0.91 (-1.46; -0.36) | | NA | | NA | |
| **Aerobic** | 1 | 2,867 | -0.20 (-0.88; 0.48) | | NA | | NA | |
| **PA + Co-intervention** | 2 | 1,855 | -0.12 (-0.51; 0.27) | | P = 0.01 | | 83.6% | |
| **Mixed PA types** | 3 | 3,135 | -0.54 (-0.70; -0.38) | | P = 0.78 | | 0% | |
| Sensitivity analysis: perinatal depressive symptoms | | | | | | | | |
| **Light-intensity PA** | 2 | 651 | | -0.72 (-1.01; -0.43) | | P = 0.36 | | 0% |
| **Moderate-intensity PA** | 1 | 155 | | -0.91 (-1.46; -0.36) | | P = | |  |
| **Vigorous-intensity PA** | N/A | N/A | | N/A | | N/A | | N/A |
| **Mixed-intensity PA** | 4 | 4,093 | | -0.45 (-0.72; -0.18) | | P = 0.08 | | 55.6% |
| **Yoga** | 3 | 967 | | -0.55 (-1.46; -0.36) | | P = 0.03 | | 70.6% |
| **Walking** | 1 | 155 | | -0.91 (-1.46; -0.36) | | NA | | NA |
| **Aerobic** | N/A | N/A | | N/A | | N/A | | N/A |
| **PA + Co-interventions** | N/A | N/A | | N/A | | N/A | | N/A |
| **Mixed PA types** | 4 | 3,403 | | -0.54 (-0.64; -0.45) | | P = 0.92 | | 0% |

## **Supplementary table S14: GRADE Evidence Profile**

References

1	Higgins JPT, Altman DG, Gøtzsche PC, et al. The Cochrane Collaboration's tool for assessing risk of bias in randomised trials. *BMJ* 2011; **343:** d5928. https://doi.org/10.1136/bmj.d5928

2	Fusar-Poli P, Radua J. Ten simple rules for conducting umbrella reviews. *Evidence-based mental health* 2018; **21:** 95–100. https://doi.org/10.1136/ebmental-2018-300014

3	Pollock M, Fernandes RM, Becker LA, Pieper D, Hartling L. Chapter V: Overviews of Reviews [last updated August 2023]. In: Higgins JPT, Thomas J, Chandler J, Cumpston M, Li T, Page MJ, Welch VA (editors), ed. Cochrane Handbook for Systematic Reviews of Interventions version 6.5. Cochrane, 2024.

4	Hemming K, Bowater RJ, Lilford RJ. Pooling systematic reviews of systematic reviews: a Bayesian panoramic meta-analysis. *Stat Med* 2012; **31:** 201–16. https://doi.org/10.1002/sim.4372

5	Schmidt FL, Oh I-S. Methods for second order meta-analysis and illustrative applications. *Organizational Behavior and Human Decision Processes* 2013; **121:** 204–18. https://doi.org/10.1016/j.obhdp.2013.03.002

6	Cooper H, Koenka AC. The overview of reviews: unique challenges and opportunities when research syntheses are the principal elements of new integrative scholarship. *The American psychologist* 2012; **67:** 446–62. https://doi.org/10.1037/a0027119

7	Türk S, Korfmacher A-K, Gerger H, van der Oord S, Christiansen H. Interventions for ADHD in childhood and adolescence: A systematic umbrella review and meta-meta-analysis. *Clinical psychology review* 2023; **102:** 102271. https://doi.org/10.1016/j.cpr.2023.102271

8	Singh B, Bennett H, Miatke A, et al. Effectiveness of exercise for improving cognition, memory and executive function: a systematic umbrella review and meta-meta-analysis. *Br J Sports Med* 2025; **59:** 866–76. https://doi.org/10.1136/bjsports-2024-108589

9	Rebar AL, Stanton R, Geard D, Short C, Duncan MJ, Vandelanotte C. A meta-meta-analysis of the effect of physical activity on depression and anxiety in non-clinical adult populations. *Health Psychol Rev* 2015; **9:** 366–78. https://doi.org/10.1080/17437199.2015.1022901

10	Pollock A, Farmer SE, Brady MC, et al. An algorithm was developed to assign GRADE levels of evidence to comparisons within systematic reviews. *Journal of Clinical Epidemiology* 2016; **70:** 106–10. https://doi.org/10.1016/j.jclinepi.2015.08.013

11	Schünemann HJ, Higgins JPT, Vist GE, Glasziou P, Akl EA, Skoetz N, Guyatt GH. Chapter 14: Completing ‘Summary of findings’ tables and grading the certainty of the evidence [last updated August 2023]. In: Higgins JPT, Thomas J, Chandler J, Cumpston M, Li T, Page MJ, Welch VA (editors), ed. Cochrane Handbook for Systematic Reviews of Interventions version 6.5. Cochrane, 2024.

12	Neumann I, Schünemann H. GRADE Book version 1.0. https://book.gradepro.org/ (accessed Oct 28, 2025).

13	Meader N, King K, Llewellyn A, et al. A checklist designed to aid consistency and reproducibility of GRADE assessments: development and pilot validation. *Syst Rev* 2014; **3:** 82. https://doi.org/10.1186/2046-4053-3-82

14	Miao C, Chen F, Chen Q, et al. Effects of different mind-body interventions for pregnant women on mental health: A systematic review and network meta-analysis of randomized controlled trials. *Journal of affective disorders* 2026; **405:** 121549. https://doi.org/10.1016/j.jad.2026.121549

15	Gong H, Ni C, Shen X, Wu T, Jiang C. Yoga for prenatal depression: a systematic review and meta-analysis. *BMC Psychiatry* 2015; **15:** 14. https://doi.org/10.1186/s12888-015-0393-1

16	Ng QX, Venkatanarayanan N, Loke W, et al. A meta-analysis of the effectiveness of yoga-based interventions for maternal depression during pregnancy. *Complementary Therapies in Clinical Practice* 2019; **34:** 8–12. https://doi.org/10.1016/j.ctcp.2018.10.016

17	Wang J, Carru C, Sedda S, Fiori PL, Li Z, Chen Z. Comparative impact of exercise-based interventions for postpartum depression: A Bayesian network meta-analysis. *International journal of gynaecology and obstetrics: the official organ of the International Federation of Gynaecology and Obstetrics* 2024; **165:** 67–75. https://doi.org/10.1002/ijgo.15091

18	Zhang Z, Liu YJ, Sun L, Zhao X-D. The effects of exercise on antenatal depression: a systematic review and meta-analysis. *Front. Psychiatry* 2024; **15:** 1290418. https://doi.org/10.3389/fpsyt.2024.1290418

19	Liu X, Wang G, Cao Y. Physical exercise interventions for perinatal depression symptoms in women: A systematic review and meta-analysis. *Front. Psychol.* 2022; **13:** 1022402. https://doi.org/10.3389/fpsyg.2022.1022402

20	Zhu Y, Wang R, Tang X, Li Q, Xu G, Zhang A. The effect of music, massage, yoga and exercise on antenatal depression: A meta-analysis. *Journal of affective disorders* 2021; **292:** 592–602. https://doi.org/10.1016/j.jad.2021.05.122

21	Morres ID, Tzouma N-A, Hatzigeorgiadis A, et al. Exercise for perinatal depressive symptoms: A systematic review and meta-analysis of randomized controlled trials in perinatal health services. *Journal of affective disorders* 2022; **298:** 26–42. https://doi.org/10.1016/j.jad.2021.10.124

22	Carter T, Bastounis A, Guo B, Jane Morrell C. The effectiveness of exercise-based interventions for preventing or treating postpartum depression: a systematic review and meta-analysis. *Arch Womens Ment Health* 2019; **22:** 37–53. https://doi.org/10.1007/s00737-018-0869-3

23	Davenport MH, McCurdy AP, Mottola MF, et al. Impact of prenatal exercise on both prenatal and postnatal anxiety and depressive symptoms: a systematic review and meta-analysis. *Br J Sports Med* 2018; **52:** 1376–85. https://doi.org/10.1136/bjsports-2018-099697

24	Hicks LE, Graf MD, Yeo S. Prenatal exercise and its effects on postpartum mental health: systematic review and meta-analysis. *Archives of women's mental health* 2024. https://doi.org/10.1007/s00737-024-01525-2

25	McCurdy AP, Boulé NG, Sivak A, Davenport MH. Effects of Exercise on Mild-to-Moderate Depressive Symptoms in the Postpartum Period: A Meta-analysis. *Obstet Gynecol* 2017; **129:** 1087–97. https://doi.org/10.1097/AOG.0000000000002053

26	Nakamura A, van der Waerden J, Melchior M, Bolze C, El-Khoury F, Pryor L. Physical activity during pregnancy and postpartum depression: Systematic review and meta-analysis. *Journal of affective disorders* 2019; **246:** 29–41. https://doi.org/10.1016/j.jad.2018.12.009

27	Pritchett RV, Daley AJ, Jolly K. Does aerobic exercise reduce postpartum depressive symptoms? a systematic review and meta-analysis. *Br J Gen Pract* 2017; **67:** e684-e691. https://doi.org/10.3399/bjgp17X692525

28	Xu H, Liu R, Wang X, Yang J. Effectiveness of aerobic exercise in the prevention and treatment of postpartum depression: Meta-analysis and network meta-analysis. *PLoS One* 2023; **18:** e0287650. https://doi.org/10.1371/journal.pone.0287650

29	Daley AJ, Foster L, Long G, et al. The effectiveness of exercise for the prevention and treatment of antenatal depression: systematic review with meta-analysis. *BJOG : an international journal of obstetrics and gynaecology* 2015; **122:** 57–62. https://doi.org/10.1111/1471-0528.12909

30	He L, Soh KL, Huang F, et al. The impact of physical activity intervention on perinatal depression: A systematic review and meta-analysis. *Journal of affective disorders* 2023; **321:** 304–19. https://doi.org/10.1016/j.jad.2022.10.026

31	Singh B, Olds T, Curtis R, et al. Effectiveness of physical activity interventions for improving depression, anxiety and distress: an overview of systematic reviews. *British journal of sports medicine* 2023; **57:** 1203–09. https://doi.org/10.1136/bjsports-2022-106195

32	Ludwig-Walz H, Siemens W, Heinisch S, Dannheim I, Loss J, Bujard M. Physical activity and physical fitness among children and adolescents after the onset of the COVID-19 pandemic in the WHO European Region: a systematic review protocol. *BMJ Open* 2023; **13:** e073397. https://doi.org/10.1136/bmjopen-2023-073397

33	ISSG Search Filters Resource. Systematic Reviews: Filters. https://sites.google.com/a/york.ac.uk/issg-search-filters-resource/home/systematic-reviews.

34	Wolters Kluwer Health, Inc. Ovid Tools and Resources Portal. https://tools.ovid.com/ovidtools/expertsearches.html.

35	Fédération des Milieux Documentaires. Périnatalité. https://extranet.santecom.qc.ca/wiki/!biblio3s/doku.php?id=concepts:perinatalite.

36	McGowan J, Sampson M, Salzwedel DM, Cogo E, Foerster V, Lefebvre C. PRESS Peer Review of Electronic Search Strategies: 2015 Guideline Statement. *Journal of Clinical Epidemiology* 2016; **75:** 40–46. https://doi.org/10.1016/j.jclinepi.2016.01.021
