# Supplementary material for: Physical activity and perinatal depression and anxiety: a global umbrella review and meta-meta-analysis
Source: eClinicalMedicine. 2026 Jul 19;98:104054. doi: 10.1016/j.eclinm.2026.104054 (PMC13523840; doi:10.1016/j.eclinm.2026.104054)
Supplement: Supplementary file 2 [file mmc2.docx]

*The following translations in French were submitted by the authors and we reproduce them as supplied. They have not been peer reviewed. Our editorial processes have only been applied to the original abstract in English, which should serve as reference for this manuscript.*

**Résumé**

**Contexte**
L’activité physique est de plus en plus reconnue comme une intervention non pharmacologique réduisant le risque de dépression et d’anxiété périnatales, bien que les résultats des méta-analyses précédentes soient hétérogènes. Cette revue de revues a synthétisée les données probantes concernant les effets globaux de l’activité physique sur la dépression et l’anxiété périnatales chez les femmes, y compris selon l’intensité et le type d’activité.

**Méthodes**
Nous avons effectué des recherches dans PubMed, Embase, Web of Science, Cochrane Library, PsycINFO, Epistemonikos, DoPHER, CINAHL et Sports Medicine & Education Index pour la littérature publiée entre janvier 2014 et avril 2026. Les revues systématiques éligibles évaluaient des essais contrôlés randomisés portant sur l’effet d’interventions d’activité physique sur la dépression et/ou l’anxiété périnatales. Des binômes d’auteurs ont réalisé l’extraction des données ainsi que les évaluations de la qualité méthodologique (AMSTAR-2) et du niveau de certitude des preuves (GRADE). Le chevauchement entre les revues systématiques incluses a été évalué à l’aide de l’aire couverte corrigée. Des méta-méta-analyses à effets aléatoires (une méta-analyse de méta-analyses) ont produit des différences moyennes standardisées (DMS) avec des intervalles de confiance à 95 % (IC à 95 %). Registre PROSPERO : CRD42024538941.

**Résultats**
Au total, 4 109 études ont été identifiées et 20 revues systématiques avec méta-analyses ont été incluses. Le chevauchement était de 8,49 %. Globalement, l’activité physique réduit les symptômes dépressifs durant la période périnatale avec un niveau de certitude modéré des preuves (DMS : -0,56 ; IC à 95 % : -0,93 à -0,19). L’activité physique d’intensité légère (DMS : -0,52 ; IC à 95 % : -0,81 à -0,23) pourrait avoir des effets comparables à ceux des interventions d’intensité modérée (DMS : -0,43 ; IC à 95 % : -0,64 à -0,22) et d’intensité mixte (DMS : -0,52 ; IC à 95 % : -0,80 à -0,24). Le yoga (DMS : -0,55 ; IC à 95 % : -0,95 à -0,15) et les activités mixtes (DMS : -0,54 ; IC à 95 % : -0,70 à -0,38) pourraient être particulièrement bénéfiques. L’activité physique pourrait également être efficace pour réduire l’anxiété (DMS : -0,85 ; IC à 95 % : -1,29 à -0,42), bien que les recherches soient plus limitées.

**Interprétation**
L’activité physique d’intensité légère et modérée est positivement associée à une réduction des symptômes de dépression et d’anxiété périnatales chez les femmes. L’activité physique devrait être recommandée comme une approche non pharmacologique, faisable et rentable pour lutter contre la dépression périnatale, et potentiellement contre l’anxiété, à l’échelle mondiale.

**Financement**
Aucun.

*The following translations in Spanish were submitted by the authors and we reproduce them as supplied. They have not been peer reviewed. Our editorial processes have only been applied to the original abstract in English, which should serve as reference for this manuscript.*

**Resumen**

**Antecedentes**

La actividad física es cada vez más reconocida como una intervención no farmacológica que reduce el riesgo de depresión y ansiedad perinatales; sin embargo, los hallazgos de metaanálisis previos son heterogéneos. Esta *umbrella review* (revision de revisiones) sintetizó la evidencia sobre los efectos generales de la actividad física en la depresión y la ansiedad perinatales en mujeres, incluyendo análisis según la intensidad y el tipo de actividad.

**Métodos**

Se realizaron búsquedas en PubMed, Embase, Web of Science, Cochrane Library, PsycINFO, Epistemonikos, DoPHER, CINAHL y Sports Medicine & Education Index de la literatura publicada entre enero de 2014 y abril de 2026. Las revisiones sistemáticas elegibles evaluaron ensayos controlados aleatorizados sobre el efecto de intervenciones de actividad física en desenlaces de depresión y/o ansiedad perinatales. Dos autores realizaron de forma independiente la extracción de datos y las evaluaciones de calidad metodológica (AMSTAR-2) y de certeza de la evidencia (GRADE). La superposición entre las revisiones sistemáticas incluidas se evaluó mediante el *corrected covered area* (el área de cobertura corregida). Los meta-meta-análisis con efectos aleatorios (metaanálisis de metaanálisis) generaron diferencias de medias estandarizadas (DME) con intervalos de confianza del 95% (IC del 95%). Registro PROSPERO: CRD42024538941.

**Resultados**

Se recuperaron un total de 4.109 estudios y se incluyeron 20 revisiones sistemáticas con metaanálisis. La superposición fue del 8∙49%. En general, la actividad física reduce los síntomas depresivos durante el período perinatal con evidencia de certeza moderada (DME: -0∙56; IC del 95%: --0∙93 a -0∙19). La actividad física de baja intensidad (DME: -0∙52; IC del 95%: -0∙81 a -0∙23) puede tener efectos comparables a los de las intervenciones de intensidad moderada (DME: -0∙43; IC del 95%: -0∙64 a -0∙22) y de intensidad mixta (DME: -0∙52; IC del 95%: -0∙80 a 0∙24). El yoga (DME: -0∙55; IC del 95%: -0∙95 a -0∙15) y las intervenciones de actividad física de intensidad mixta (DME: -0∙54; IC del 95%: -0∙70 a -0∙38) pueden ser particularmente beneficiosos. La actividad física también puede ser eficaz para reducir la ansiedad (DME: -0∙85; IC del 95%: -1∙29 a -0∙42), aunque la investigación es más limitada.

**Interpretación**

La actividad física de baja intensidad y de intensidad moderada se asocia con una reducción de los síntomas de depresión y ansiedad perinatales en mujeres. La actividad física debería recomendarse como un enfoque no farmacológico, factible y coste-efectivo para abordar la depresión perinatal y, potencialmente, la ansiedad a nivel mundial.

**Financiación**

Ninguna.

*The following translations in German were submitted by the authors and we reproduce them as supplied. They have not been peer reviewed. Our editorial processes have only been applied to the original abstract in English, which should serve as reference for this manuscript.*

**Zusammenfassung**

**Hintergrund**
Körperliche Aktivität wird zunehmend als nicht-pharmakologische Intervention anerkannt, die das Risiko für perinatale Depressionen und Angststörungen verringern kann. Die Ergebnisse früherer Meta-Analysen sind jedoch uneinheitlich. Dieses Umbrella Review analysiert die Evidenz zum Effekt körperlicher Aktivität auf perinatale Depressionen und Angststörungen bei Frauen, einschließlich der Auswirkungen unterschiedlicher Intensitäten und Arten körperlicher Aktivität.

**Methoden**
Wir durchsuchten PubMed, Embase, Web of Science, die Cochrane Library, PsycINFO, Epistemonikos, DoPHER, CINAHL sowie den Sports Medicine & Education Index nach Literatur, die zwischen Januar 2014 und April 2026 veröffentlicht wurde. Eingeschlossen wurden systematische Reviews, die randomisierte kontrollierte Studien zur Wirkung körperlicher Aktivitätsinterventionen auf perinatale Depressions- und/oder Angststörungssymptome untersuchten. Die Datenextraktion sowie die Bewertung der methodischen Qualität (AMSTAR-2) und der Vertrauenswürdigkeit der Evidenz (GRADE) wurden jeweils paarweise von den Autoren und Autorinnen durchgeführt. Überlappungen zwischen den eingeschlossenen systematischen Reviews wurden mithilfe des Corrected Covered Area (CCA) bewertet. Standardisierte Mittelwertsdifferenzen (SMDs) mit 95-%-Konfidenzintervallen (95%-KI) wurden mit Random-Effects-Meta-Meta-Analysen (Meta-Analysen von Meta-Analysen) berechnet. PROSPERO-Registrierung: CRD42024538941.

**Ergebnisse**
Insgesamt wurden 4.109 Studien identifiziert; 20 systematische Reviews mit Meta-Analysen wurden eingeschlossen. Das CCA betrug 8,49 %. Insgesamt reduziert körperliche Aktivität depressive Symptome während der Perinatalzeit mit moderater Vertrauenswürdigkeit der Evidenz (SMD: −0,56; 95-%-KI: −0,93 bis −0,19). Körperliche Aktivität von geringer Intensität (SMD: −0,52; 95-%-KI: −0,81 bis −0,23) könnte vergleichbare Effekte wie Interventionen mittlerer Intensität (SMD: −0,43; 95-%-KI: −0,64 bis −0,22) und gemischter Intensität (SMD: −0,52; 95-%-KI: −0,80 bis −0,24) haben. Yoga (SMD: −0,55; 95-%-KI: −0,95 bis −0,15) und gemischte Arten der Aktivität (SMD: −0,54; 95-%-KI: −0,70 bis −0,38) könnten besonders vorteilhaft sein. Körperliche Aktivität könnte zudem auch bei der Verringerung von Angststörungen wirksam sein (SMD: −0,85; 95-%-KI: −1,29 bis −0,42). Allerdings ist die Evidenz dazu begrenzt.

**Interpretation**
Leichte und moderate körperliche Aktivität sind positiv mit einer Verringerung perinataler depressiver und Angstsymptome bei Frauen assoziiert. Körperliche Aktivität sollte weltweit als nicht-pharmakologischer, umsetzbarer und kosteneffektiver Ansatz zur Behandlung perinataler Depressionen, und möglicherweise auch zur Behandlung von Angststörungen, empfohlen werden.

**Finanzierung**
Keine.

*The following translations in Chinese were submitted by the authors and we reproduce them as supplied. They have not been peer reviewed. Our editorial processes have only been applied to the original abstract in English, which should serve as reference for this manuscript.*

**摘要**

**背景**
体力活动日益被认为是一种可降低围产期抑郁和焦虑风险的非药物干预措施，但既往荟萃分析的结果存在异质性。本伞状综述综合评估了体力活动对女性围产期抑郁和焦虑总体影响的证据，并进一步分析了不同活动强度和类型的效果。

**方法**
我们检索了 PubMed、Embase、Web of Science、Cochrane Library、PsycINFO、Epistemonikos、DoPHER、CINAHL 及 Sports Medicine & Education Index 中于2014年1月至2026年4月期间发表的文献。纳入的系统综述均评估了随机对照试验中体力活动干预对围产期抑郁和/或焦虑结局的影响。两位作者独立完成数据提取、方法学质量评估（AMSTAR-2）及证据确定性评估 (GRADE）。采用校正覆盖面积法评估纳入系统综述之间的重叠程度。采用随机效应 meta-meta 分析（即荟萃分析的荟萃分析）计算标准化均数差（SMD）及其95%置信区间（95% CI）。PROSPERO 注册号：CRD42024538941。

**结果**
共检索到4,109项研究，最终纳入20项包含荟萃分析的系统综述。综述间重叠率为8∙49%。总体而言，体力活动可降低围产期抑郁症状，且证据确定性为中等（SMD：−0∙56；95% CI：−0∙93 至 −0∙19）。低强度体力活动（SMD：−0∙52；95% CI：−0∙81 至 −0∙23）的效果可能与中等强度（SMD：−0∙43；95% CI：−0∙64 至 −0∙22）及混合强度干预（SMD：−0∙52；95% CI：−0∙80 至 −0∙24）相当。瑜伽（SMD：−0∙55；95% CI：−0∙95 至 −0∙15）及混合类型活动（SMD：−0∙54；95% CI：−0∙70 至 −0∙38）可能尤其有益。体力活动也可能有效降低焦虑水平（SMD：−0∙85；95% CI：−1∙29 至 −0∙42），但相关研究仍较有限。

**解释**
低强度和中等强度体力活动与女性围产期抑郁和焦虑症状的减少呈正相关。体力活动应被推荐作为一种非药物、可行且具有成本效益的方法，用于全球范围内应对围产期抑郁，并可能有助于缓解围产期焦虑。

**资金来源**
无。
